# Supplementary material for: The role of APOBEC3B in lung tumor evolution and targeted cancer therapy resistance
Source: Nat Genet. 2023 Dec 4;56(1):60–73. doi: 10.1038/s41588-023-01592-8 (PMC10786726; doi:10.1038/s41588-023-01592-8)
Supplement: Supplementary file 1 — Supplementary Note. [file 41588_2023_1592_MOESM1_ESM.pdf]

nature genetics

Article

https://doi.org/10.1038/s41588-023-01592-8

The role of APOBEC3B in lung tumor evolution and targeted cancer therapy resistance

In the format provided by the authors and unedited

Supplementary Note (Main points raised by Reviewers during all rounds of peer-review)

To highlight the controversies in the field, and to outline the limitations of our experimental approach we have included this discussion of all the main points raised by the Reviewers over the 2.5-year peer-review process at both *Nature* and *Nature Genetics*. This summary aims to provide a balanced and scholarly discussion that contributes to moving the field forward.

Note: Any data/figures not included in the manuscript and discussed here have been included below and referenced in the text (Fig. X herein).

Reviewer comments categorized:

1. **Genetically engineered mouse models**
2. ***In vitro* experimental work to confirm A3 induction with targeted therapy**
3. **Function of A3 family members in targeted therapy resistance development**
4. **Human patient datasets**

1. Genetically engineered mouse models

- *The Reviewers asked why there is no difference in survival between EA3B mice and E control mice (Fig. 1a, herein) despite there being increased tumor cell death in EA3B mice at tumor initiation (Fig. 1e, manuscript).*

We hypothesized that as tumours progress, additional alterations like loss of p53 pathway activation increases tolerance for *A3B* expression. We quantified p53 nuclear expression levels using immunohistochemical (IHC) staining and observed a significant increase in p53 nuclear positivity in tumours in *EA3B* mice at 3 months post induction (Extended Data Fig. 1c, manuscript, Early timepoint). Next, we examined levels of p53 activation by IHC in mice at termination (Late timepoint) and found that there was no longer a significant difference in p53 positivity (Extended Data Fig. 1d, manuscript). We also examined p53 pathway activation at this late timepoint using qPCR of p53 targets on DNA harvested from tumours of both *E* and *EA3B* mice. No significant difference in p53 pathway activation was observed (Fig. 1b-g, herein). These data together suggest p53 pathway activation could be required for the increased tumor cell death observed at tumor initiation.

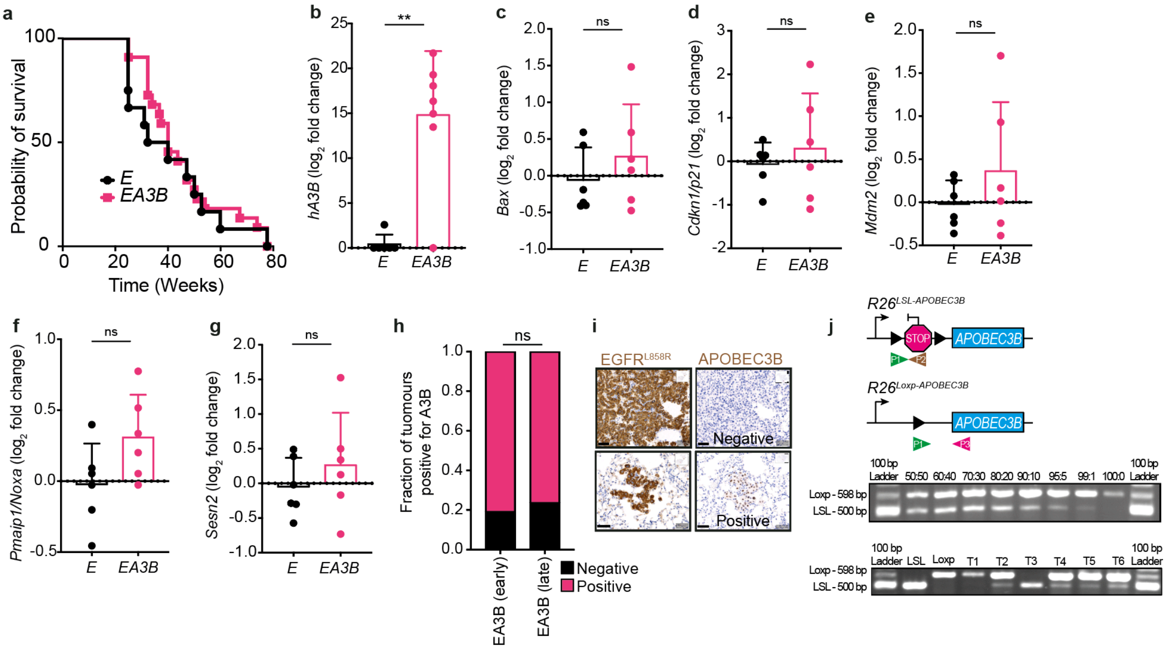

Fig. 1: No Increase in CIN or p53 activation is observed with continuous A3B expression late in tumor evolution. **a**, Survival curve of *E* versus *EA3B* mice (*E*=15, *EA3B* = 24, each dot represents a mouse). **b**, *A3B* expression in *E* and *EA3B* tumors at termination (*E*=6, *EA3B* =7, $\log_2$ fold change). **c,** *Bax* expression in *E* and *EA3B* tumors at termination (*E*=6, *EA3B* =7, $\log_2$ fold change, *A3B* negative tumor in EA3B mouse identified in b removed from subsequent analysis of p53 activation). **d**, *Cdkn1/p21* expression in *E* and *EA3B* tumors at termination (*E*=6, *EA3B* =7, $\log_2$ fold change, *A3B* negative tumor in EA3B mouse identified in b removed from subsequent analysis of p53 activation). **e,** *Mdm2* expression in *E* and *EA3B* tumors at termination (*E*=6, *EA3B* =7, $\log_2$ fold change, *A3B* negative tumor in EA3B mouse identified in b removed from subsequent analysis of p53 activation). **f**, *Pmaip1/Noxa* expression in *E* and *EA3B* tumors at termination (*E*=6, *EA3B* =7, $\log_2$ fold change, *A3B* negative tumor in EA3B mouse identified in b removed from subsequent analysis of p53 activation). **g**, *Sesn2* expression in *E* and *EA3B* tumors at termination (*E*=6, *EA3B* =7, $\log_2$ fold change, *A3B* negative tumor in EA3B mouse identified in b removed from subsequent analysis of p53 activation). **h**, Fraction of tumors positive for A3B at early and late timepoints measured by IHC staining (Early E =3, EA3B =3, Late E = 8, EA3B =8). **i**, Representative IHC staining of EGFRL858R, and APOBEC3B. Scale bar =50 µm. **j,** PCR analysis of recombination efficiency of LoxP-Stop-LoxP site upstream of human APOBEC3B transgene. Primer 1 (P1) in LoxP, Primer 2 (P2) in stop codon, Primer 3 (P3) in human APOBEC3B transgene. P1+P2 = LSL 500 bp, P1+P3 = 598 bp. EA3B tumors at a late timepoint (termination). LSL = unrecombined tail DNA, Loxp = fully recombined cell line DNA. Ratios represent fraction of unrecombined tail DNA to fully recombined cell line DNA.

To illustrate that inactivation of the p53 pathway is sufficient to blunt the tumor cell death observed at initiation with *A3B* expression, we showed that in a p53-deficient mouse model, there is no difference in tumour number at 3 months post-induction between *EP* and *EPA3B* mice (Fig. 1k-l, manuscript). There was also no difference in overall survival between *EP* and *EPA3B* mice (Fig. 1m, manuscript). These data suggest that from tumour initiation A3B expression is tolerated in a p53-deficient background.

Finally, to rule out that the lack of difference in survival between *E* and *EA3B* mice was not due to loss of A3B expression, we quantified A3B levels in tumours at 3 months post-initiation and at termination by immunohistochemical (IHC) staining and saw no significant difference (Fig. 1h-i, herein) We also performed PCR to assess recombination in six tumours harvested at termination from *EA3B* mice. Five out of six of these tumours had a recombination efficiency above 90%, and one tumour was unrecombined. This rate of recombination (5/6) aligned with

the rate of recombination we observed by IHC staining at 3 months and at termination (Fig. 1 j, herein).

The Reviewers expressed concerns that because the mouse models contain a human A3B transgene (Fig. 1a, manuscript), an immune response to the A3B transgene could be driving the increased tumor cell death observed – rather than due to A3B expression and activity.

We performed the following experiments to address the Reviewers' comments. First, we induced tumors in an $EGFR^{L858R}$ mutant, APOBEC3B (A3B) cytidine deaminase inactive (*E(CAG)A3B*^{E255A}) mouse model and compared the $EGFR^{L858R}$ positive cells per lung lobe per mouse in this model compared to $EGFR^{L858R}$ control mice (Fig. 1h-j, manuscript). Unlike in wildtype A3B mice, in A3B cytidine deaminase inactive mice no significant difference in $EGFR^{L858R}$ positive cells per lung area was observed compared to control mice at the same early timepoint (Fig. 1i, manuscript). This suggests that the cytidine deaminase activity of A3B is partially contributing to the tumor cell death observed with A3B expression at tumor initiation, but also that the human transgene itself is likely not contributing to an immune response.

To explore this further, we also performed intravenous transplantation of *EPA3B* mouse tumor cell lines generated from tumors in pure C57BL/6J *EPA3B* genetically engineered mouse models (GEMMs). These cell lines were transplanted intravenously into either wildtype C57BL/6 mice or *EPA3B* GEMM C57BL/6J mice (Extended Data Fig. 1j-m, manuscript). Note that the *EPA3B* GEMM C57BL/6J mice were uninduced mice that do not express the $EGFR^{L858R}$ or APOBEC3B transgenes. We observed tumors that stained positively for $EGFR^{L858R}$ and A3B at 4 weeks and 12 weeks post-intravenous injection in the *EPA3B* C57BL/6J mice, but no tumors at both timepoints in wildtype C57BL/6J mice (Extended Data Fig. 1j-m, manuscript). This suggests that the *EPA3B* C57BL/6 mice are at least somewhat tolerized to both the $EGFR^{L858R}$ and A3B transgenes. We also don't yet know if the $A3B^{E255A}$ form of A3B retains enzyme independent effects, such as driving increased chromosomal instability, which could also alter the tumor immune microenvironment. Further exploration of the tumor immune microenvironment in *E(CAG)A3B*^{E255A} mutant mice, and use of immunodeficient mouse models to fully characterize this immune tolerance to human *EGFR*^{L858R} and *APOBEC3B* transgenes will be crucial in future work.

The Reviewers asked if, in the mouse models where the induction of A3B was temporally separated from the induction of the oncogenic transgene $EGFR^{L858R}$ *resulting in subclonal expression of A3B (EA3Bi) (Extended Data Fig. 2a, manuscript), in the absence of targeted therapy is there a difference in survival between mice expressing subclonal A3B (EA3Bi) and control mice (E)?*

In response, we set up a survival experiment where subclonal A3B was induced in the absence of TKI therapy. Survival was compared in untreated *E* and *EA3Bi* mice and a significant increase in survival of A3B-expressing *EA3Bi* mice compared with *E* control mice which lack A3B (Extended Data Fig. 2a-d, manuscript). At termination we stained lung sections for $EGFR^{L858R}$ and A3B. Subclonal A3B positive tumor cells were confirmed in *EA3Bi* mice. We next compared the level of A3B positivity between tumours in TKI-treated *EA3Bi* to untreated *EA3Bi* mice and found a significant increase in A3B positivity with TKI (Fig. 3f, manuscript). This suggests that

with the administration of TKI therapy there is a selection for A3B expression. Subsequently, we worked with two independent pathologists from the Royal Veterinary College who assessed tumour nodules per lung section per mouse and tumour area per lung section per mouse in untreated *E* mice compared with *EA3Bi* mice, and observed a significantly higher number of tumour nodules and tumour area per lung section in *E* control mice compared with *EA3Bi* mice at termination (Extended Data Fig. 2a-c, manuscript). These findings suggests that subclonal expression of A3B, is detrimental for tumorigenesis and tumor growth at initiation.

2. *In vitro* experimental work to confirm A3 induction with targeted therapy

The Reviewers requested immunofluorescent (IF) experiments to determine if tyrosine kinase inhibitor (TKI) treatment leads to a change in distribution (cytoplasmic versus nuclear) of RelB.

As per the Reviewer recommendation, we further validated the increase in nuclear RELB upon TKI treatment through an IF assay. Consistent with the results of our western blot analysis, we find that treatment of PC9 cells for 1 day with the EGFR inhibitor osimertinib leads to increased nuclear RELB levels (Fig. 2 a-b, herein).

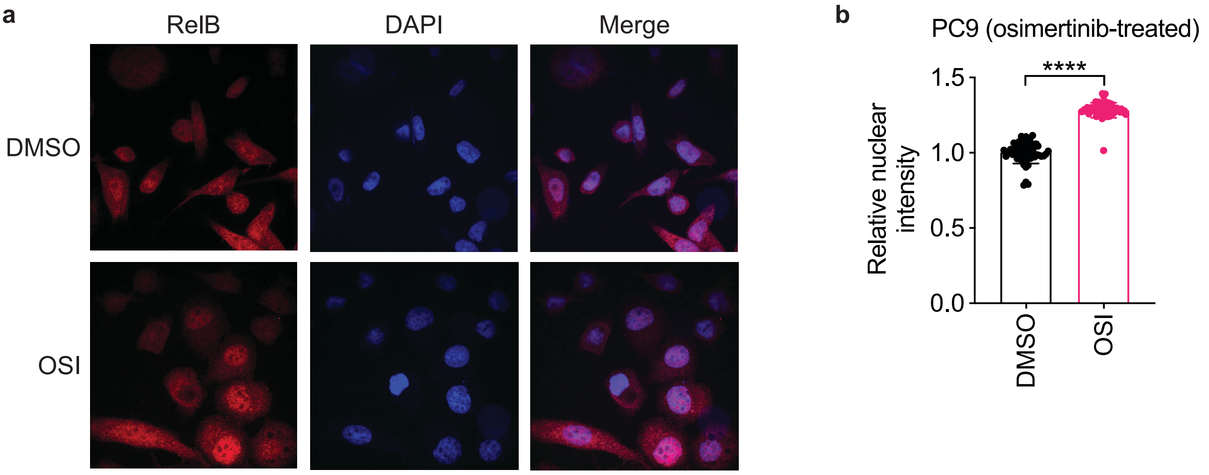

Fig. 2: Assessment of nuclear RELB levels using immunofluorescence assay. a, Representative images of immunofluorescence assay to examine nuclear RelB levels in PC9 cells treated with DMSO or 1 μM osimertinib (osi). **b**, Quantification of nuclear RelB levels in PC9 cells treated from part a (n = 3 biological replicates, mean ± SEM, ANOVA test, ****P < 0.0001).

The Reviewers proposed that the authors should treat EGFR mutant cell lines with EGFR TKI alone, NF-κB inhibitor (PBS-1086) alone, or the combination, and assess progression free survival and A3B induction.

First, we performed the experiment requested by the Reviewers and examined A3B expression and found that co-treatment with PBS-1086 blocks the induction of A3B that occurs upon EGFR TKI treatment (Extended Fig. 8a, manuscript). We also showed in a previously published study[1] that co-treatment with PBS-1086 is more effective than EGFR inhibitor treatment alone at preventing the emergence of resistance. These data provide support for the causal role of NF-κB pathway in driving the evolution of resistance under TKI therapy.

__The Reviewers asked if the induction of A3B through NF-κB signaling is specific to targeting the mutant oncoprotein, or is it more generalized to any drug with cytotoxic effects like chemotherapy.__

We confirmed that the induction of A3B upon TKI treatment was conserved across multiple oncoprotein inhibitors of the same general class – i.e., inhibitors of oncogenic receptor tyrosine kinase enzymes (RTK) – and is likely not a consequence of an off-target effect of EGFR or ALK inhibitors. To address this, we tested and found similar induction of *A3B* using two different EGFR and ALK inhibitors (EGFR inhibitors: erlotinib and osimertinib; ALK inhibitors: crizotinib and alectinib) (Fig. 5a-e and Extended Data Fig. 6a-e, manuscript).

We also observed induction of *A3B* upon treatment with inhibitors of mitogen-activated protein kinase enzymes MEK1 and/or MEK2 (MEK inhibitor), selumetinib/AZD6244 (Fig. 5a). We confirmed the specificity of these effects by determining if APOBEC induction occurs upon genetic depletion of the driver oncoprotein by siRNA treatment. We found that *A3B* induction occurs upon genetic depletion of EGFR, in an oncogenic EGFR-driven lung adenocarcinoma model (Fig. 5f, manuscript).

Induction of A3B and APOBEC activity has also been shown to occur upon treatment of cancer cells with chemotherapeutic drugs, likely through DNA replication stress[2,3], but no correlation was observed between drug-induced cytotoxicity and APOBEC3 activity[2]. Chemotherapy-induced transcriptional upregulation of A3B has been shown to be dependent on NF-κB activation[3,4]. We observed an induction of *A3B* expression upon treatment with chemotherapeutic agent (gemcitabine) in an EGFR mutant cell line (Fig. 3, herein). Our data also show that the NF-κB pathway contributes to *A3B* induction (Extended Data Fig. 8, manuscript). Altogether, these results suggest that APOBEC induction could occur upon treatment with chemotherapeutic drugs, potentially through NF-κB activation, and is likely not merely a consequence of general cytotoxicity.

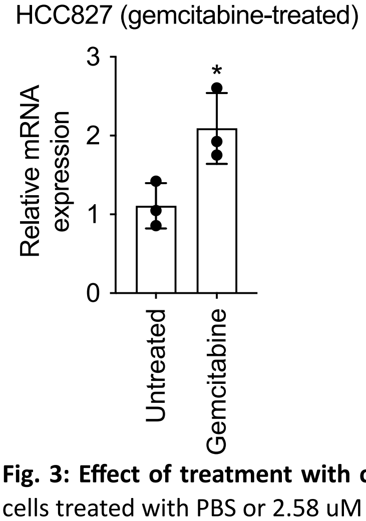

Fig. 3: Effect of treatment with chemotherapeutic drug on APOBEC expression. RT-qPCR analysis of HCC827 cells treated with PBS or 2.58 uM Gemcitabine (cytotoxic chemotherapy) and assayed in triplicate (n=3, mean ± SD, two-tailed t-test, *P=0.033)

__The Reviewers requested that the analysis of the effects of EGFR TKI osimertinib on A3B expression was expanded to physiological as well as supraphysiological doses of both osimertinib and erlotinib.__

The plasma concentration of osimertinib at the 80 mg standard dose is below 1 μM[5]. According to the FDA (#208065Orig1s000), in healthy volunteers, the C_{max} at the 80 mg dose was 126 nM. We performed A3B expression analyses using multiple EGFR mutant lung cancer cell lines and observed significant increases in A3B expression in all three cell lines with physiological (0.1 μM and 0.5 μM) (Fig. 4a-c, herein) and supraphysiological dosing (above 1 μM) (Fig. 5, manuscript).

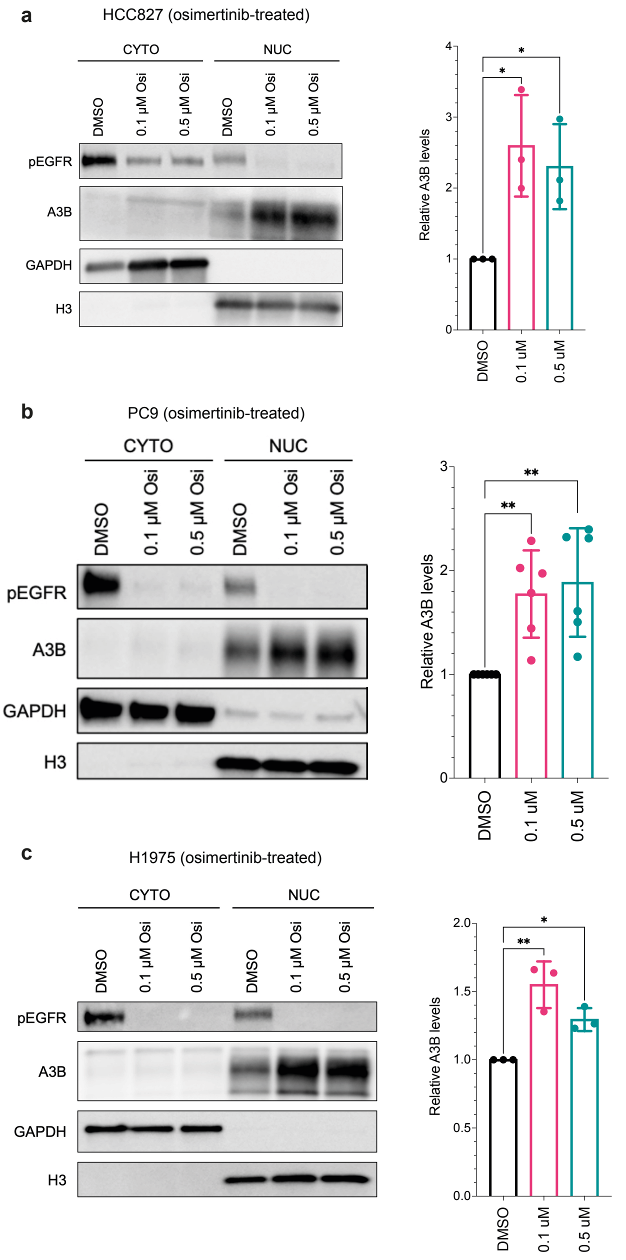

Fig. 4: Osimertinib induces A3B expression in multiple EGFR mutant lung cancer cell lines. a, Immunoblotting for A3B in HCC827 lung cancer cells treated with 0.1 μM or 0.5 μM osimertinib with quantification (n = 3 biological replicates, mean ± SD, one-way Anova test, *P = 0.0219, *P = 0.0253). b, Immunoblotting for A3B in PC9 lung cancer cells treated with 0.1 μM or 0.5 μM osimertinib with quantification (n = 6 biological replicates, mean ± SD, one-way Anova test, **P = 0.0065, **P = 0.0024). c, Immunoblotting for A3B in H1975 lung cancer cells treated with 0.1 μM or 0.5 μM osimertinib with quantification (n = 3 biological replicates, mean ± SD, one-way Anova test, **P = 0.0017, *P=0.0169).

The Reviewers asked if there is a clear connection between the increase in A3B protein and upregulation of A3B mRNA, or if alternative post translational modifications might play a role.

We examined if *A3B* mRNA and protein levels correlate in two different EGFR mutant lung cancer cell lines. In the first cell line (PC9) the levels of *A3B* mRNA and protein increased simultaneously with osimertinib treatment (Fig. 5a-b, herein). In the other cell line (HCC827) there was also a significant increase in *A3B* mRNA and protein levels, although mRNA expression levels were slightly higher than protein levels observed (Fig. 5c-d, herein). This may be due to delayed A3B protein synthesis[6] in HCC827 cells, with a more rapid increase in mRNA transcription after TKI treatment compared with protein translation, or to differences in post-translational modifications of A3B in PC9 cells versus HCC827 cells.

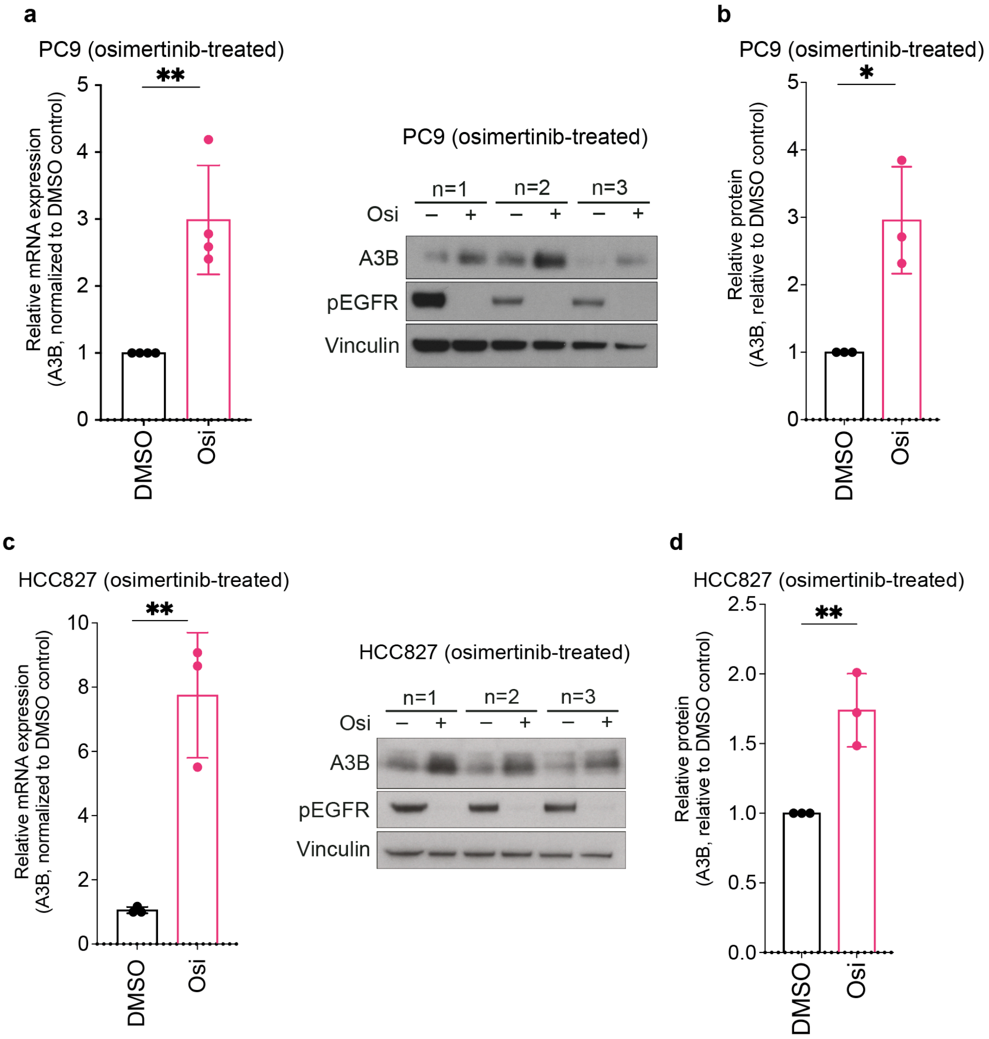

Fig. 5: *APOBEC3B* mRNA and protein levels correlate well with physiological dose of TKI osimertinib. a, RT-qPCR performed on PC9 cells treated with DMSO or 0.5 uM osimertinib for 18 hours (n = 4 biological replicates, mean ± SD, t-test, **P = 0.0027). b, Western blot analysis for A3B protein levels in PC9 cells treated with DMSO or 0.5 uM osimertinib for 18 hours (n= 3 biological replicates, mean ± SD, t-test, *P = 0.0129). c, RT-qPCR performed on HCC827 cells treated with DMSO or 0.5 uM osimertinib for 18 hours (n = 3 biological replicates, mean ± SD, t-test, **P = 0.0040). d, Western blot analysis for A3B protein levels in HCC827 cells treated with DMSO or 0.5 uM osimertinib for 18 hours (n= 3 biological replicates, mean ± SD, t-test, **P = 0.0082).

Limited work has been done on post-translational modifications of A3B by intrinsic cellular factors, but it is known that the serine/threonine kinase, protein kinase A (PKA), binds to A3B and phosphorylates Thr214, disrupting binding between the A3B catalytic core and ssDNA, inhibiting its activity[7]. Another more recent finding demonstrated that the interleukin enhancer-binding factor 2 (ILF2) can interact with A3B and increase its deaminase activity while in its high molecular mass complex in the nucleus[8]. We believe post-translational modifications affecting protein stability of A3B likely impacts mRNA and protein correlations, an area for future study.

The Reviewers asked for analysis of the expression levels and activity of multiple APOBEC3 family members including A3A, A3B, and A3F in multiple lung cancer cell lines treated with TKIs and with or without knockdown of each of the APOBEC3 family members included.

We performed these experiments and observed that in PC9 cells, the expression of all four APOBEC3 family members increased significantly with osimertinib treatment, although *A3A* expression was increased to the greatest extent (Fig. 4a, manuscript). In HCC827 cells, all four APOBEC3 family members examined were also significantly increased with osimertinib treatment, with both *A3A* and A3B being increased to the greatest extent and to a similar level (Fig. 4b, manuscript). We also obtained data illustrating that with osimertinib treatment there was a significant increase in APOBEC activity in both PC9 and HCC827 cells (Fig. 4c-d, manuscript).

We examined how knockdown of each of the four APOBEC3 family members affected the APOBEC catalytic activity induced with TKI treatment. In both PC9 and HCC827 cells, only knockdown of A3B resulted in a significant reduction of APOBEC activity induced with TKI treatment (Fig. 4g, manuscript). These findings have been independently verified in an additional preprint from another research group[9]. Our findings highlight the general role of APOBEC induction in response to targeted therapy and suggest that A3B along with other family members should be studied further by all investigators.

3. Function of A3 family members in targeted therapy resistance development

The Reviewers stated that the observation that A3B can contribute to resistance in mice does not imply that is also does so in human cancers where other A3s are operative. The Reviewers then requested that experiments in cancer cell lines treated with targeted therapy over clinically relevant timeframes be performed to assess if A3B is critical for resistance development and if this resistance occurs through A3B-mediated mutagenesis.

To address the Reviewer’s comments, additional experiments in A3B-proficient and -deficient human cancer cell lines treated over more clinically relevant time frames with the TKI osimertinib were performed. First A3B was knocked out in three lung cancer cell lines: two EGFR mutant, PC9 and HCC827, and one ALK fusion model, H3122 (Extended Data Fig. 4a-i, manuscript). In the case of PC9 and HCC827 cells, the parental control (sgGFP) and knockout (sgA3B) lines were then single cell cloned (Extended Data Fig. 4a-d, manuscript). None of the knockout cell lines showed off-target effects on other APOBEC3 family members (Extended Data Fig. 4a-f, manuscript) or differences in cell viability when treated with DMSO over 7 days (Extended Data Fig. 4g-I,manuscript). Cell viability was compared with TKI treatment

(osimertinib for PC9 and HCC827 and alectinib for H3122) over a 12-week timecourse experiment. Significant reductions in cell viability were observed in A3B-deficient cells compared to A3B-proficient cells in all three cancer cell lines with long-term TKI treatment (Fig. 3j-l, manuscript). Knockout of A3B had no effect on cell viability in untreated PC9, HCC827 or H3122 cell lines (Extended Data Fig. 4g-i, manuscript).

To further address the Reviewer's comments regarding whether A3B is required for the accumulation of APOBEC single base substitution signatures (SBS2+13), A3B-deficient and -proficient single-cell cloned PC9 cells (Extended Data Fig. 4a-b, manuscript) were treated with osimertinib using a dose escalation protocol until resistance, for a total continuous treatment time of 3 months (Fig. 6a, manuscript). The mutations acquired over the treatment time course were assessed through whole genome sequencing (WGS) analysis and the proportion of APOBEC mutation signatures (SBS2+13) acquired was quantified (Fig. 6b and Extended Data Fig. 9a, manuscript). This revealed that only A3B-proficient lines had enrichment of APOBEC signatures SBS2+13 during TKI treatment, while A3B-deficient lines did not (Fig. 6b, manuscript). Examination of the fraction of mutations in an APOBEC context (TCW C>T/G) revealed a significant decrease in A3B-deficient lines (Fig. 6c, manuscript). Examination of APOBEC pentanucleotide sequences[10-13] in the osimertinib-treated A3B-deficient and A3B-proficient groups (Fig. 6d-e, manuscript) revealed significant decreases in the fraction of APOBEC mutations in an A3B-preferred RTCW context in A3B-deficient clones with no significant decrease in mutations in a A3A-preferred YTCW context (Fig. 6d-e, manuscript). These data support the role of A3B in the enrichment of APOBEC signature mutations SBS2+13 during TKI treatment. Along with the previous observation that absence of A3B reduces APOBEC activity and also cell viability under targeted therapy (Fig. 3j-l, manuscript), these data suggest that one of the mechanisms through which A3B could contribute to resistance is increased APOBEC mutagenesis.

Although initial findings in the field suggest that APOBEC-associated pentanucleotide sequences (A3A-YTCW and A3B-RTCW) can distinguish between A3A and A3B enzymatic activity[10-12], emerging evidence suggests that in certain contexts, for instance, in one example a human chronic myelogenous leukemia (CML) cell line[13], A3B can display a YTCW bias, illustrating that using these pentanucleotide sequences to interpret whether A3A- or A3B-driven mutagenesis has occurred remains controversial.

However it is important to note that A3B is likely not the only source of mutagenesis leading to targeted therapy resistance. In fact, as the Reviewers mention, other A3 family members, such as A3A, have recently been shown to contribute to the development of targeted therapy resistance mutations[14]. Therefore, while we show evidence of A3B being a source of mutagenesis with TKI treatment, it is one of multiple possible mechanisms of resistance to targeted therapy such as activation of alternate pathways, dysregulation of downstream targets and histological transformation, which is consistent with emerging themes noted in the revised manuscript.

The Reviewers point out that in knockdown and knockout experiments in human cancer cell lines expression of other APOBEC3 family members should be quantified to ensure that knock-

outs/downs are specific to A3B, given the highly homologous sequences of APOBEC3 family members, which may contribute to examined phenotypes.

We used three A3B CRISPR knockout human lung cancer cell lines for the long-term cell viability experiments (Extended Data Fig. 4a-i, manuscript) and for our long-term TKI treatment A3B knockout whole genome sequencing experiment (Fig. 6a, manuscript). The sgRNA sequence targeting A3B was obtained from the Zhang lab's GeCKO v2 libraries of sgRNAs, which were designed using improved calculations of off-target scores based on specificity analysis to have as minimal off-target effects as possible[15]. This sgRNA was used for all knockout cell lines.

We verified that the knockout lines are specific to A3B by assessing mRNA expression levels of all *APOBEC3* family members compared to control lines and observed significant reduction of *A3B* expression but no significant difference in expression of any of the other *APOBEC3* family members in A3B knockout lines compared with A3B-proficient controls (Extended Data Fig. 4a-f, manuscript).

For the shA3B knockdown and re-expression experiments verifying that the increased APOBEC activity observed with TKI treatment is driven specifically by the catalytically active form of A3B (Extended Data Fig. 6j-l, manuscript), we verified the specificity of the shRNA targeting A3B by assessing mRNA expression levels of all other *APOBEC3* family members. No off-target reductions in any other *APOBEC3* family members were observed (Extended Data Fig. 6m, manuscript). Interestingly, significant increases in *A3A*, *A3G*, and *A3H* expression were detected (Extended Data Fig. 6m, manuscript). Increases in A3A expression and protein stabilization with A3B knockdown have been previously shown in human breast and lymphoma cancer cell lines[11], but this is the first time an increase in *A3A* expression with A3B knockdown has been reported in a human lung cancer cell line. Despite this induction of other A3 family members upon A3B suppression, we observed significant reduction of APOBEC activity, long-term cell viability under TKI therapy, and loss of enrichment of APOBEC signatures (2+13) under TKI therapy in our A3B-deficient lung cancer cell lines.

The Reviewers stated that the links between targeted therapy resistance and A3B are indirect, and that to uncover the relevant mechanism by which A3B contributes to resistance the catalytic domain of endogenous A3B in a human cell line would have to beinactived and resistance/mutation acquisition would have to be examined.

We agree that an experiment with the catalytic domain of A3B perturbed would be important to directly link A3B-mediated mutagenesis as the mechanism by which A3B contributes to resistance. This was not however the question we set out to answer in our manuscript, which was more centered on highlighting the complex and context-dependent role of A3B in lung cancer. We noted in the discussion of our manuscript the limitations of our study and the alternative enzymatic independent mechanisms of resistance such as the increased chromosomal instability observed with A3B expression, cell cycle regulation[16] and DNA damage repair pathway regulation[17,18] that could be contributing to TKI resistance. We also highlighted additional experiments requested by the Reviewers to strengthen our data illustrating how A3B deficiency in human cancer cell line models reduces both cell viability over clinically relevant timeframes (Fig. 3l-m, manuscript), APOBEC mutation signature

(Sig2+13) acquisition and enrichment with TKI therapy treatment (Fig. 6b-g, manuscript), and acquisition in post-TKI clinical samples of mutations in APOBEC context in genes previously implicated in TKI resistance (Fig. 7g, manuscript).

The Reviewers stated that they were interested in the authors thoughts on the temporal dynamics of APOBEC3 induction, specifically why might there be a lag between induction of APOBEC3 expression and activity and resistance. Could this be due to the time it takes for subclonal outgrowth or another factor?

We agreed with the Reviewer that this was an interesting question that was worth discussing. We observed APOBEC3 upregulation early after treatment with TKI therapies; this APOBEC3 induction could allow for the acquisition of mutations during TKI treatment. Mutations acquired may confer a survival benefit in the presence of the drug (resistance mutations) or may not confer any benefit in the presence of the drug (passenger or non-consequential mutations). Furthermore, as the Reviewer suggested it would also take time for the resistant subclones to outgrow. Thus, the delay could be due to both: 1] time needed to acquire a resistance mutation that aids cancer cells in evading targeted therapy and is then selected for during therapy and 2] time needed for selection of the resistance mutation and clones. Resistance will of course arise due to mechanisms independent of APOBEC activity. Our study suggests that A3B upregulation is one mechanism driving the evolution of resistance under targeted therapy, but it is certainly not the only mechanism.

4. Human patient datasets

The Reviewers expressed concerns about our patient datasets including that the numbers of matched patients are small, and that upregulation of A3B does not occur in all samples which should be discussed.

These clinical specimens are a unique resource and one that is very challenging to acquire and analyse. Over the course of our revisions we obtained 17 additional samples from patients to increase the numbers in our bulk RNA-sequencing dataset, for a total of 74 samples in our study. Analysis of all patient samples in this dataset demonstrated a significant increase in A3B expression, with a greater significant increase than our original analysis. The changes in expression pre- and post-TKI of all *APOBEC3* family members were also assessed. With this analysis, we uncovered that only *A3B* is significantly upregulated with TKI treatment. Stratifying by treatment timepoints revealed a significant increase from treatment naïve (TN) to residual disease (RN) and an increase approaching significance from TN to progressive disease (PD). The observation that not all samples show *A3B* induction is important and accurate, and not unexpected given the heterogeneity present in patients with NSCLC[19] and the multitude of evolutionary pathways to targeted therapy resistance[20].

To further support our findings of overall increased *A3B* expression post-TKI we analyzed a single-cell RNA-sequencing dataset collected during targeted therapy[21]. We annotated cancer cells with EGFR and ALK mutations treated with TKIs and examined *A3B* expression in patients with TN, RD, and PD tumors treated with targeted therapy. We observed a significant increase in APOBEC3 family member expressions, with *A3B* having the second highest increase effect

size when comparing *A3B* expression between TN and PD and RD and PD stages of all APOBEC3 family members in this single cell RNA-seq dataset (A3C had the highest increase effect size, however APOBEC activity assays revealed A3C did not contribute to overall activity with TKI treatment).

The Reviewers questioned if the A3B upregulation observed in patient samples is potentially due to chemotherapy treatment versus TKI therapy specifically.

To address this question, we stratified our expanded clinical sequencing cohort (bulk RNA-sequencing dataset) into patients treated with single agent TKI or patients who had received both TKI + chemo. We observed significant increases of *A3B* in patients who had received single agent TKI as well as in patients who had received TKI + chemo, indicating that TKI therapy alone is sufficient for the clinical association with increased *A3B* expression (Fig. 6, herein). Recent findings suggest that chemotherapy can also induce *A3B* expression[22], which could explain why we observe increased *A3B* expression (perhaps modestly higher) in patient tumors treated with both TKI and chemotherapy, beyond the TKI therapy alone (Fig. 6, herein).

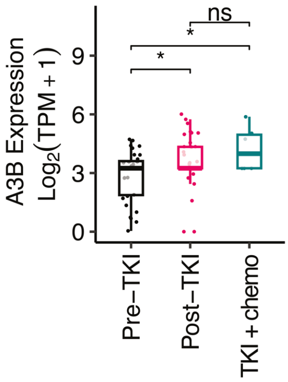

Fig. 6: *APOBEC3B* expression increases significantly with TKI therapy with or without chemotherapy in patients with non-small cell lung carcinoma (NSCLC). Comparison of *APOBEC3B* expression levels (Exp: batch corrected TPM) measured using RNA-seq analysis in human NSCLC specimens obtained before pre or post treatment with single agent TKI (Post-TKI) or with a combination of TKI and chemotherapy (TKI+chemo) from lung cancer patients undergoing treatment with TKI (all data points shown, one-way ANOVA test, mixed effects model, *P=0.033, *P=0.029).

References:

1 Blakely, C. M. *et al.* NF-κB-activating complex engaged in response to EGFR oncogene inhibition drives tumor cell survival and residual disease in lung cancer. *CellReports* **11**, 98-110, doi:10.1016/j.celrep.2015.03.012 (2015).

2 Kanu, N. *et al.* DNA replication stress mediates APOBEC3 family mutagenesis in breast cancer. *Genome Biol* **17**, 185, doi:10.1186/s13059-016-1042-9 (2016).

3 Periyasamy, M. *et al.* Induction of APOBEC3B expression by chemotherapy drugs is mediated by DNA-PK-directed activation of NF-kappaB. *Oncogene*, doi:10.1038/s41388-020-01583-7 (2020).

4 Leonard, B. *et al.* The PKC/NF-κB signaling pathway induces APOBEC3B expression in multiple human cancers. *Cancer Research* **75**, 4538-4547, doi:10.1158/0008-5472.CAN-15-2171-T (2015).
5 Pilla Reddy, V., Walker, M., Sharma, P., Ballard, P. & Vishwanathan, K. Development, Verification, and Prediction of Osimertinib Drug-Drug Interactions Using PBPK Modeling Approach to Inform Drug Label. *CPT Pharmacometrics Syst Pharmacol* **7**, 321-330, doi:10.1002/psp4.12289 (2018).
6 Gedeon, T. & Bokes, P. Delayed protein synthesis reduces the correlation between mRNA and protein fluctuations. *Biophys J* **103**, 377-385, doi:10.1016/j.bpj.2012.06.025 (2012).
7 Matsumoto, T. *et al.* Protein kinase A inhibits tumor mutator APOBEC3B through phosphorylation. *Sci Rep* **9**, 8307, doi:10.1038/s41598-019-44407-9 (2019).
8 Kazuma, Y. *et al.* ILF2 enhances the DNA cytosine deaminase activity of tumor mutator APOBEC3B in multiple myeloma cells. *Sci Rep* **12**, 2278, doi:10.1038/s41598-022-06226-3 (2022).
9 Garcia, N. M. G. *et al.* APOBEC3 activity promotes the survival and evolution of drug-tolerant persister cells during acquired resistance to EGFR inhibitors in lung cancer. *bioRxiv*, doi:10.1101/2023.07.02.547443 (2023).
10 Petljak, M. *et al.* Characterizing Mutational Signatures in Human Cancer Cell Lines Reveals Episodic APOBEC Mutagenesis. *Cell* **176**, 1282-1294 e1220, doi:10.1016/j.cell.2019.02.012 (2019).
11 Petljak, M. *et al.* Mechanisms of APOBEC3 mutagenesis in human cancer cells. *Nature* **607**, 799-807, doi:10.1038/s41586-022-04972-y (2022).
12 Chan, K. *et al.* An APOBEC3A hypermutation signature is distinguishable from the signature of background mutagenesis by APOBEC3B in human cancers. *Nat Genet* **47**, 1067-1072, doi:10.1038/ng.3378 (2015).
13 Jarvis, M. C. *et al.* Mutational impact of APOBEC3B and APOBEC3A in a human cell line. *biorxiv.org*, doi:10.1101/2022.04.26.489523 (2022).
14 Isozaki, H. *et al.* Therapy-induced APOBEC3A drives evolution of persistent cancer cells. *Nature* **620**, 393-401, doi:10.1038/s41586-023-06303-1 (2023).
15 Sanjana, N. E., Shalem, O. & Zhang, F. Improved vectors and genome-wide libraries for CRISPR screening. *Nature Methods* **11**, 783-784, doi:10.1038/nmeth.3047 (2014).
16 McCann, J. L. *et al.* The DNA deaminase APOBEC3B interacts with the cell-cycle protein CDK4 and disrupts CDK4-mediated nuclear import of Cyclin D1. *J Biol Chem* **294**, 12099-12111, doi:10.1074/jbc.RA119.008443 (2019).
17 Nikkila, J. *et al.* Elevated APOBEC3B expression drives a kataegic-like mutation signature and replication stress-related therapeutic vulnerabilities in p53-defective cells. *Br J Cancer* **117**, 113-123, doi:10.1038/bjc.2017.133 (2017).
18 Buisson, R., Lawrence, M. S., Benes, C. H. & Zou, L. APOBEC3A and APOBEC3B Activities Render Cancer Cells Susceptible to ATR Inhibition. *Cancer Res* **77**, 4567-4578, doi:10.1158/0008-5472.CAN-16-3389 (2017).
19 Jamal-Hanjani, M. *et al.* Tracking the Evolution of Non-Small-Cell Lung Cancer. *The New England journal of medicine*, doi:10.1056/NEJMoa1616288 (2017).
20 Rotow, J. & Bivona, T. G. Understanding and targeting resistance mechanisms in NSCLC. *Nat Rev Cancer* **17**, 637-658, doi:10.1038/nrc.2017.84 (2017).

21 Maynard, A. *et al.* Therapy-Induced Evolution of Human Lung Cancer Revealed by Single-Cell RNA Sequencing. *Cell* **182**, 1232-1251.e1222, doi:10.1016/j.cell.2020.07.017 (2020).

22 Periyasamy, M. *et al.* Induction of APOBEC3B expression by chemotherapy drugs is mediated by DNA-PK-directed activation of NF-kappaB. *Oncogene* **40**, 1077-1090, doi:10.1038/s41388-020-01583-7 (2021).
