# Supplementary material for: The role of APOBEC3B in lung tumor evolution and targeted cancer therapy resistance
Source: Nat Genet. 2023 Dec 4;56(1):60–73. doi: 10.1038/s41588-023-01592-8 (PMC10786726; doi:10.1038/s41588-023-01592-8)
Supplement: Supplementary file 2 — Reporting Summary [file 41588_2023_1592_MOESM2_ESM.pdf]

nature portfolio

Corresponding author(s): Trever G. Bivona and Deborah R. Caswell

Last updated by author(s): 10/11/2023



# Reporting Summary

Nature Portfolio wishes to improve the reproducibility of the work that we publish. This form provides structure for consistency and transparency in reporting. For further information on Nature Portfolio policies, see our Editorial Policies and the Editorial Policy Checklist.

## Statistics

For all statistical analyses, confirm that the following items are present in the figure legend, table legend, main text, or Methods section.

| n/a | Confirmed | |
|---|---|---|
| ☐ | ☒ | The exact sample size (*n*) for each experimental group/condition, given as a discrete number and unit of measurement |
| ☐ | ☒ | A statement on whether measurements were taken from distinct samples or whether the same sample was measured repeatedly |
| ☐ | ☒ | The statistical test(s) used AND whether they are one- or two-sided<br>*Only common tests should be described solely by name; describe more complex techniques in the Methods section.* |
| ☐ | ☒ | A description of all covariates tested |
| ☐ | ☒ | A description of any assumptions or corrections, such as tests of normality and adjustment for multiple comparisons |
| ☐ | ☒ | A full description of the statistical parameters including central tendency (e.g. means) or other basic estimates (e.g. regression coefficient) AND variation (e.g. standard deviation) or associated estimates of uncertainty (e.g. confidence intervals) |
| ☐ | ☒ | For null hypothesis testing, the test statistic (e.g. *F*, *t*, *r*) with confidence intervals, effect sizes, degrees of freedom and *P* value noted<br>*Give P values as exact values whenever suitable.* |
| ☒ | ☐ | For Bayesian analysis, information on the choice of priors and Markov chain Monte Carlo settings |
| ☒ | ☐ | For hierarchical and complex designs, identification of the appropriate level for tests and full reporting of outcomes |
| ☐ | ☒ | Estimates of effect sizes (e.g. Cohen's *d*, Pearson's *r*), indicating how they were calculated |

*Our web collection on statistics for biologists contains articles on many of the points above.*

## Software and code

Policy information about availability of computer code

| | |
|---|---|
| Data collection | GraphPad Prism 7, QuantStudio 12K Flex Software V1.3, Adobe Illustrator 26.2.1, ImageJ-2, Microsoft Excel, AnalyzeDirect, QuPath v0.1.2, RSEM (version 1.2.29), VarScan2(v2.4.1), MuTect(v1.1.7) and Scalpel(v0.5.4), PROVEAN (Protein Variation Effect Analyzer), GATK bundle (v2.8), GATK3 (version 3.6.0), R (version 3.3.1) and deepSNV (v1.18.1), Burrows-Wheeler Aligner (version 0.7.17), Picard (version 2.18.16), deconstructSigs R, MuTect (v1.1.6), Oncotator, Strelka (v1.0.11), deTiN, DESeq, GSEA. |
| Data analysis | Cell line Whole Genome Mutational Signature Analysis:<br>Sequences were aligned to the human genome (hg38) using the Burrows-Wheeler Aligner (version 0.7.17). PCR duplicates were removed using Picard (version 2.18.16). Reads were locally realigned around InDels using GATK3 (version 3.6.0) tools RealignerTargetCreator to create intervals, followed by IndelRealigner on the aligned bam files. MuTect2 from GATK3 (version 3.6.0) was used in tumour/normal mode to call mutations in test vs control cell lines. Single nucleotide variants (SNVs) that passed the internal GATK3 filter with read depths over 30 reads at called positions, at least 4 reads in the alternate mutation call and an allele frequency greater than 0.1 were used for downstream analysis. Figures were plotted using the deconstructSigs R package (Rosenthal et al. 2016).<br><br>Mutation analysis:<br>Paired-end reads were aligned to the hg19 human genome using the Picard pipeline (https://gatk.broadinstitute.org/). A modified version of the Broad Institute Getz Lab CGA WES Characterization pipeline (https://docs-google-com.ezp-prod1.hul.harvard.edu/document/d/1VO2kX_fgfUd0x3mBS9NjLUWGZu794WbTepBel3cBg08) was used to call, filter and annotate somatic mutations. Specifically, single-nucleotide variants (SNVs) and other substitutions were called with MuTect (v1.1.6) (Cibulskis et al., 2013). Mutations were annotated using Oncotator (Ramos et al., 2015). MuTect mutation calls were filtered for 8-OxoG artifacts, and artifacts introduced through the formalin fixation process (FFPE) of tumour tissues (Costello et al., 2013). Indels were called with Strelka (v1.0.11). MuTect calls and Strelka calls were further filtered through a panel of normal samples (PoN) to remove artifacts generated by rare error modes and miscalled germline |

alterations 65. To pass quality control, samples were required to have <5% cross-sample contamination as assessed with ContEst (Cibulskis et al., 2013); mean target coverage of at least 25x in the tumour sample and 20x in the corresponding normal as assessed using GATK3.7 DepthOfCoverage; and a percentage of tumour-in-normal of < 30% as determined by deTiN 68. This pipeline was modified for analysis of cell lines rather than tumour-normal pairs as follows: indels were called through MuTect2 alone rather than Strelka; deTiN was not performed; and a common variant filter was applied to exclude variants present in The Exome Aggregation Consortium (ExAC) if at least 10 alleles containing the variant were present across any subpopulation, unless they appeared in a list of known somatic sites (Lek et al. 2016 and AACR Project Genei Consortium, 2017).

Mutational signature analysis:
Active mutational processes (Alexandrov et al., 2013) were determined using the deconstructSigs R package, with a signature contribution cutoff of 6%. This cutoff was chosen because it was the minimum contribution value required to obtain a false-positive rate of 0.1% and false-negative rate of 1.4% per the authors' in-silico analysis, and is the recommended cutoff (Rosenthal et al., 2016). Samples with < 10 mutations were excluded from analysis due to poor signature discrimination with so few mutations.

RNA-Seq analyses: PDX-tissue RNA extractions were carried using RNeasy micro kit (Qiagen). RNA-Seq was performed using replicate samples on the Illumina HiSeq4000, paired-end 100-bp reads at the Center for Advanced Technology (UCSF). For the differential gene expression analysis, DESeq program was used to compare controls to erlotinib samples as previously described (Anders & Huber, 2010).
RNAseq samples form patients and cell lines were sequenced by Novogene (https://en.novogene.com/) with paired-end sequencing (150bp in length). There were ~20 million reads for each sample. The processed fastq files were mapped to hg19 reference genome using STAR (version 2.4) algorithm and transcript expressions were quantified using RSEM (version 1.2.29) algorithm. The default parameters in the algorithms were used. The normalized transcript reads (TPM) were used for downstream analysis.
For single cell RNA Seq analyses the data from a previously published study (excluding samples from the neoadjuvant osimertinib dataset) was used and analyzed in a similar manner (Maynard et al., 2020). All cells used are identified as malignant by marker expression and CNV inference and originated in from various biopsy sites (adrenal, liver, lymph node, lung, pleura/pleural fluid). Nonparametric, pairwise comparisons (Wilcoxon Rank Sum Test) was used to determine the statistical significance of the pairwise comparisons of different timepoints for their average scaled expression.

Human EGFR transgene amplicon sequencing of mouse: FASTQ files were aligned to hg19 obtained from the GATK bundle (v2.8) using bwa mem (bwa v0.7.15)98,99. Analyses were performed using R (version 3.3.1) and deepSNV (v1.18.1)100. The median depth of coverage of sequenced EGFR exons (19,20,21) was 5290x (range: 2238-8040). Variants associated with resistance to EGFR tyrosine kinase inhibitors were queried using deepSNV's bam2R function, with the arguments q=20 & s=2. The variants explored include: T790M, D761Y, L861Q, G796X, G797X, L792X, L747S. L858R was identified in every sequenced sample.

Whole exome sequencing – mouse data: WES was performed by the Advanced Sequencing Facility at The Francis Crick Institute using the Twist BioScience Human Core Exon Kit for library preparation and Agilent SureSelectXT Mouse All Exon, 16, Kit for library preparation respectively. Sequencing was performed on HiSeq 4000 platforms.
RNA sequencing – mouse data: RNA-seq was performed by the Advanced Sequencing Facility at the Francis Crick Institute using the KAPA mRNA HyperPrep Kit (KK8581 – 96 Libraries) and KAPA Dual-Indexed Adapters (Roche-KK8720). Sequencing was performed on HiSeq 4000 platforms. The processed fastq files were mapped to mm10 reference genome using STAR (version 2.4) algorithm and transcript expressions were quantified using RSEM (version 1.2.29) algorithm with the default parameters. The read counts were used for downstream analysis.
Alignment – mouse: All samples were de-multiplexed and the resultant FASTQ files aligned to the mm10 mouse genome, using bwa mem (bwa v0.7.15). De-duplication was performed using Picard (v2.1.1) (http://broadinstitute.github.io/picard). Quality control metrics were collated using FASTQC (v0.10.1 - http://www.bioinformatics.babraham.ac.uk/projects/fastqc/), Picard and GATK (v3.6). SAMtools (v1.3.1) was used to generate mpileup files from the resultant BAM files. Thresholds for base phred score and mapping quality were set at 20. A threshold of 50 was set for the coefficient of downgrading mapping quality, with the argument for base alignment quality calculation being deactivated. The median depth of coverage for all samples was 92x (range: 58-169x).
Variant detection & annotation – mouse: Variant calling was performed using VarScan2(v2.4.1), MuTect(v1.1.7) and Scalpel(v0.5.4)91-93. The following argument settings were used for variant detection using VarScan2:
--min-coverage 8 --min-coverage-normal 10 --min-coverage-tumor 6 --min-var-freq 0.01 --min-freq-for-hom 0.75 --normal-purity 1 --p-value 0.99 --somatic-p-value 0.05 --tumor-purity 0.5 --strand-filter 0
For MuTect, only "PASS" variants were used for further analyses. With the exception of allowing variants to be detected down to a VAF of 0.001, default settings were used for Scalpel insertion/deletion detection.
To minimise false positives, additional filtering was performed. For single-nucleotide variants (SNVs) or dinucleotides detected by VarScan2, a minimum tumor sequencing depth of 30, variant allele frequency (VAF) of 5%, variant read count of 5, and a somatic p-value <0.01 were required to pass a variant. For variants detected by VarScan2 between 2 and 5% VAF, the mutation also needs to be detected by MuTect.
As for insertions/deletions (INDELs), variants need to be passed by both Scalpel ("PASS") and VarScan2 (somatic p value <0.001). A minimum depth of 50x, 10 alt reads and VAF of 2% was required.
For all SNVs, INDELs and dinucleotides, any variant also detected in the paired germline sample with more than 5 alternative reads or a VAF greater than 1% was filtered out.
The detected variants were annotated using Annovar94.
Functional annotation of SNVs – mouse: Murine gene mutation callings from whole exome sequencing were parsed with some modification including genomic coordinates (removing 'chr' before chromosomal numbers, only 'SNV' was selected). The modified files were fed into PROVEAN (Protein Variation Effect Analyzer)95-97 software tool (http://provean.jcvi.org/index.php) to predict whether an amino acid substitution has an impact on the biological function of a protein (SIFT score). The predict files were merged with original files at gene level annotation using R program.

For manuscripts utilizing custom algorithms or software that are central to the research but not yet described in published literature, software must be made available to editors and reviewers. We strongly encourage code deposition in a community repository (e.g. GitHub). See the Nature Portfolio guidelines for submitting code & software for further information.

# Data

Policy information about availability of data

All manuscripts must include a data availability statement. This statement should provide the following information, where applicable:

- Accession codes, unique identifiers, or web links for publicly available datasets
- A description of any restrictions on data availability
- For clinical datasets or third party data, please ensure that the statement adheres to our policy

The Whole Exome Sequencing (WES) data and RNAseq data (from the TRACERx study) generated, used or analysed during this study are available through the Cancer Research UK and University College London Cancer Trials Centre (ctc.tracerx@ucl.ac.uk) for academic non-commercial research purposes only, and subject to review of a project proposal that will be evaluated by a TRACERx data access committee, entering into an appropriate data access agreement and subject to any applicable ethical approvals. The Whole Genome Sequencing (WGS) data shown in Fig. 6 will be available upon request, subject to evaluation by corresponding authors. For the single cell RNA-Seq analyses shown in Ext. Data Fig. 10b-c, the data from a previously published study (all advanced lung cancer cell data) were used and analyzed in a similar manner31. This data is available as an NCBI Bioproject # PRJNA591860. The RNA-Seq data for Ext. Data Fig. 10a was from a previously published study1. These data are available at NCBI GEO under accession number GSE65420. Related RNA-Seq and WES sequencing data will be deposited into NCBI GEO and SRA database.

# Research involving human participants, their data, or biological material

Policy information about studies with human participants or human data. See also policy information about sex, gender (identity/presentation), and sexual orientation and race, ethnicity and racism.

| | |
|---|---|
| Reporting on sex and gender | Participant sex (biological attribute) provided in supplementary tables. No sex- and gender-based analysis was performed, not relevant and outside of scope of current study. |
| Reporting on race, ethnicity, or other socially relevant groupings | Participant race provided in supplementary tables. No race-based analysis was performed, not relevant and outside of scope of current study. |
| Population characteristics | Population characteristics relevant in this study:<br>- Age (adult over 18)<br>- Lung cancer histology<br>- Oncogenic driver mutation<br>- Treatment history<br>- Treatment response<br><br>Other population characteristics provided but not relevant in this study:<br>- Smoking history |
| Recruitment | Patients were recruited according to Institutional Review Board-approved protocols CC13-6512 and CC17-658, NCT03433469. No bias to report. |
| Ethics oversight | All patients gave informed consent for collection of clinical correlates, tissue collection, research testing under Institutional Review Board-approved protocols listed above. Patient demographics are listed in Supplemental Tables. Patient studies were conducted according to the Declaration of Helsinki, the Belmont Report, and the U.S. Common Rule. |

Note that full information on the approval of the study protocol must also be provided in the manuscript.

# Field-specific reporting

Please select the one below that is the best fit for your research. If you are not sure, read the appropriate sections before making your selection.

☒ Life sciences ☐ Behavioural & social sciences ☐ Ecological, evolutionary & environmental sciences

For a reference copy of the document with all sections, see nature.com/documents/nr-reporting-summary-flat.pdf

# Life sciences study design

All studies must disclose on these points even when the disclosure is negative.

| | |
|---|---|
| Sample size | Sample size of clinical data was determined by access and analysis of all available cases that met criteria. Sample size of mouse experiments were determined based on previous work done using these same models (E.C. de Bruin et al. 2014), and based on previous work in the laboratory. |
| Data exclusions | Details of exclusion criteria based are described in detail in the methods section. |
| Replication | Studies involving pre-clinical models were performed with two or more biological and/or technical replicates. |

| | |
|---|---|
| Randomization | Mice were assigned to vehicle or treatment groups for xenograft studies to ensure even distribution in both groups based on the tumor size just prior to the initiation of treatment. Genetically modified mice were placed in groups based on their genetic background. |
| Blinding | Investigators were blinded during data collection, analysis of mouse tissues, and microCT analysis. |

# Reporting for specific materials, systems and methods

We require information from authors about some types of materials, experimental systems and methods used in many studies. Here, indicate whether each material, system or method listed is relevant to your study. If you are not sure if a list item applies to your research, read the appropriate section before selecting a response.

## Materials & experimental systems

| n/a | Involved in the study | |
|---|---|---|
| ☐ | ☒ | Antibodies |
| ☐ | ☒ | Eukaryotic cell lines |
| ☒ | ☐ | Palaeontology and archaeology |
| ☐ | ☒ | Animals and other organisms |
| ☐ | ☒ | Clinical data |
| ☒ | ☐ | Dual use research of concern |
| ☒ | ☐ | Plants |

## Methods

| n/a | Involved in the study | |
|---|---|---|
| ☒ | ☐ | ChIP-seq |
| ☒ | ☐ | Flow cytometry |
| ☒ | ☐ | MRI-based neuroimaging |

# Antibodies

Antibodies used

Antibodies used for western Blot analysis: pEGFR (CST-3777 or 2236), pERK (CST-4370 or 9106), APOBEC3B (A gift from Harris Lab, Brown et al., 2019), UNG (A gift from Harris Lab, Serebrenik et al., 2019), GAPDH (sc-59540), Histone H3 (CST-9715), EGFR (CST-4267), TUBB (CST-2146), Hsp90 (CST-4874), RELA (CST-8242), RELB (CST- 4922),ERK (CST- 9102), pSTAT3 (CST-9145), STAT3 (CST-9139), AKT (CST-2920) and pAKT (CST-4060).

Antibodies used for IHC analysis:
EGFRL858R mutant specific (Cell Signaling: 3197, 43B2)
APOBEC3B (5210-87-13, Brown et al,.2019)
Ki67 (Abcam: Ab15580)
Caspase 3 (R&D (Bio-Techne): AF835)
p-Histone H2AX (Sigma-Aldrich, 05-636),
UNG (NB600-1031, Novus Biologicals)
p53 (Leica, cat # NCL-L-p53-CM5p)
phospho-HIstone H3 (Sigma-Aldrich, Ser10)

Validation

Validation related to western blot analysis: APOBEC3B, UNG antibody was validated with RNAi and/or CRISPR-Cas9 mediated approaches with orthogonal validation using RT-qPCR approach. All other antibodies were validated with RNAi mediated knockdown approaches or based on the expected changes in those proteins upon treatment with inhibitors. Most of the antibodies used in this study have been extensively used in previously published studies from our laboratory (Blakely et al., 2015, Hrustanovic et al., 2015).

Validation related to IHC analysis: All of the antibodies, except p53 were previously optimized and validate in the Experimental Histopathology Unit (EHP) at the Francis Crick Institute. p53 has been validated extensively in the Vousden Laboratory and Attardi Laboratories, and by Leica Biosystems.

APOBEC3B and UNG were optimized using mouse tissue and cell lines that served as positive and negative controls. The optimization of APOBEC3B was based off of the publication Brown et al., 2019. The optimization of UNG was done using information from Novus Biologicals (https://www.novusbio.com/products/ung-antibody_nb600-1031#datasheet) and from previous publications using this antibody (Yang et al., 2005.)

# Eukaryotic cell lines

Policy information about cell lines and Sex and Gender in Research

| | |
|---|---|
| Cell line source(s) | Cell lines were purchased from ATCC as described in the previous studies from our laboratory (Blakely et al., 2015, Hrustanovic et al., 2015). Mouse tumour cell lines were generated from tumours of genetically engineered mouse models mentioned in the manuscript, and verified by flow cytometry analysis and gene expression analysis. |
| Authentication | Cell lines were previously validated by STR analysis. |
| Mycoplasma contamination | Cell lines were tested for mycoplasma contamination. |
| Commonly misidentified lines (See ICLAC register) | N/A |

## Animals and other research organisms

Policy information about studies involving animals; ARRIVE guidelines recommended for reporting animal research, and Sex and Gender in Research

| | |
|---|---|
| Laboratory animals | For xenograft studies 6-8 week old female NOD/SCID mice were used.<br><br>For GEM model studies:<br>Briefly, all mice were purified to the C57BL/6J through back crossing of at least 8 generations or purity was assessed using the a C57BL/6J substrain panel.<br><br>Genotypes of mice used in the study:<br>- TetO-EGFRL858R;R26tTA/+ (E)<br>- TetO-EGFRL858R;R26tTA/LSL-APOBEC3B (EA3B)<br>- TetO-EGFRL858R;CCSP-rtTA:R26LSL-APOBEC3B/Ert2-Cre (EA3Bi)<br><br>An approximately equal number of males and females were used in each arm of the study. Mice were housed with up to 5 mice per cage and separated by sex. Feed and water were available ad libitum.<br><br>Diet information:<br>- Regular diet: 2018s Global Rodent 18% autoclavable diet<br>- Doxycycline diet (625 ppm (Harlan-Tekland) Irradiated diet)<br><br>Water information: Mice have free access to RO water supplied by automatic watering system.<br><br>Caging information: Techniplast green line IVC cages<br><br>Chamber environment information: Mice are housed on aspen wood chip, and each cage is provided with nesting material, a mouse house and mouse loft for environmental enrichment.<br><br>Type and frequency of observation: Mice were checked once daily. Animals in each experiment were weighed initially and then weekly for the duration of the experiment. Mice were<br><br>Humane endpoints (clinical signs or situations that determine the end of the experiment for any animal under this experiment to prevent unnecessary suffering):<br><br>Mice which showed any one of the signs below were culled by an overdose of anaesthetic and then a terminal bleed (exsanguination) was performed.<br><br>If weight loss reaches 10% of original body weight within 24 hours, or 15% weight loss within 48 hours, or 15-20% over a longer period<br>• Persistent hunched posture/piloerection and laboured breathing<br>• Diarrhoea<br>• If ulceration or infection are noted<br>• If persistent self-induced trauma is noted |
| Wild animals | N/A |
| Reporting on sex | For genetically engineered mouse models, both male and female animals were used equally. For xenograft mouse models, only female NOD/SCID mice were used according to previously established UCSF IACUC-approved animal protocols. No sex-based analysis was performed, not relevant and outside of scope of current study. |
| Field-collected samples | N/A |
| Ethics oversight | The xenograft studies were approved and overseen by the UCSF IRB. All GEM model animal regulated procedures were approved by the Francis Crick Institute BRF Strategic Oversight Committee that incorporates the Animal Welfare and Ethical Review Body and conformed with the UK Home Office guidelines and regulations under the Animals (Scientific Procedures) Act 1986 including Amendment Regulations 2012. All xenograft models were conducted under UCSF IACUC-approved animal protocols. |

Note that full information on the approval of the study protocol must also be provided in the manuscript.

## Clinical data

Policy information about clinical studies

All manuscripts should comply with the ICMJE guidelines for publication of clinical research and a completed CONSORT checklist must be included with all submissions.

| | |
|---|---|
| Clinical trial registration | NCT03433469 |
| Study protocol | https://clinicaltrials.gov/study/NCT03433469#study-plan |
| Data collection | Patients were recruited for this study from July 1 2018 to August 1, 2020. Data were collected for this study at UCSF from July 1, |

| | |
|---|---|
| Data collection | 2018 to December 1, 2020. |
| Outcomes | The primary outcome of this study is to evaluate the efficacy of osimertinib as neoadjuvant therapy in patients with surgically resectable EGFR-mutant NSCLC. The primary endpoint of the study will be MPR rate defined as ≤10% viable tumor present histologically in the resected tumor specimen. Secondary measures of efficacy are radiographic decrease in maximum tumor diameter, 5-year DFS, 5-year OS, pathological response rate (pCR), and depth of response (DpR). Exploratory endpoints are to evaluate genomic and transcriptional changes on baseline and resected tumor specimens as determined by whole exome sequencing and RNA sequencing. |
