# Supplementary material for: The role of APOBEC3B in lung tumor evolution and targeted cancer therapy resistance
Source: Nat Genet. 2023 Dec 4;56(1):60–73. doi: 10.1038/s41588-023-01592-8 (PMC10786726; doi:10.1038/s41588-023-01592-8)

Fig. 4e

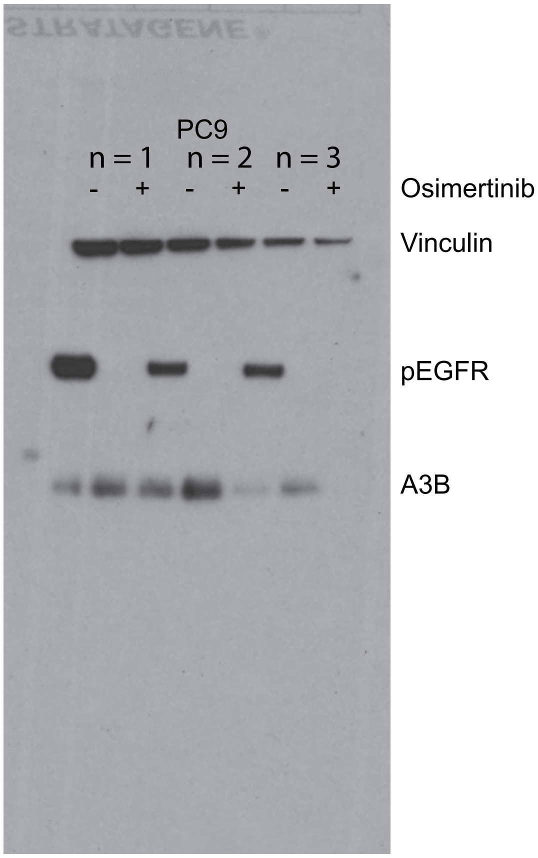

Fig. 4f

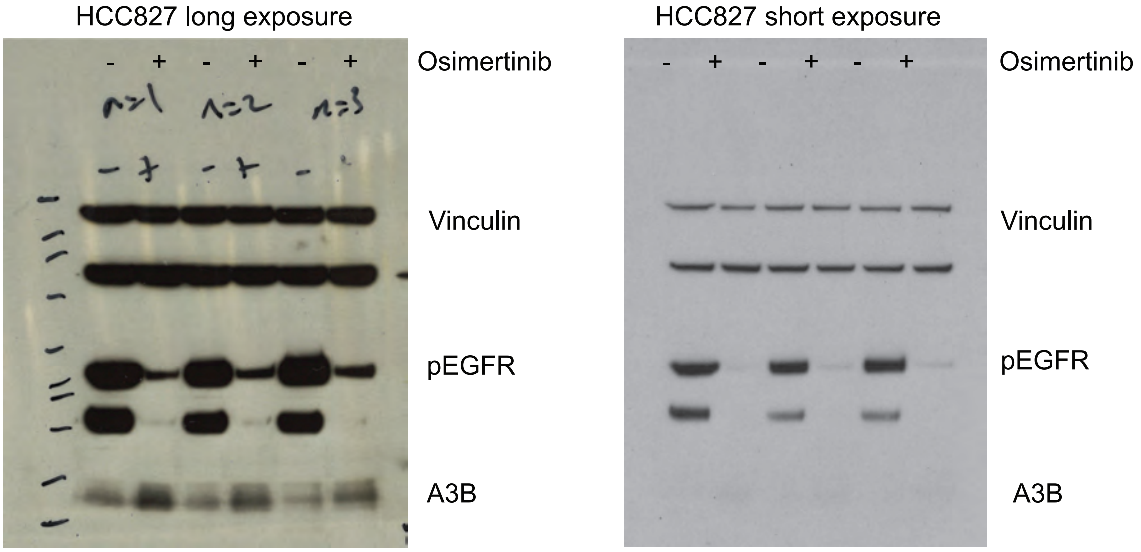
HCC827 long exposure
- + - + - +
Osimertinib
Vinculin
pEGFR
A3B
HCC827 short exposure
- + - + - +
Osimertinib
Vinculin
pEGFR
A3B

Fig. 4g

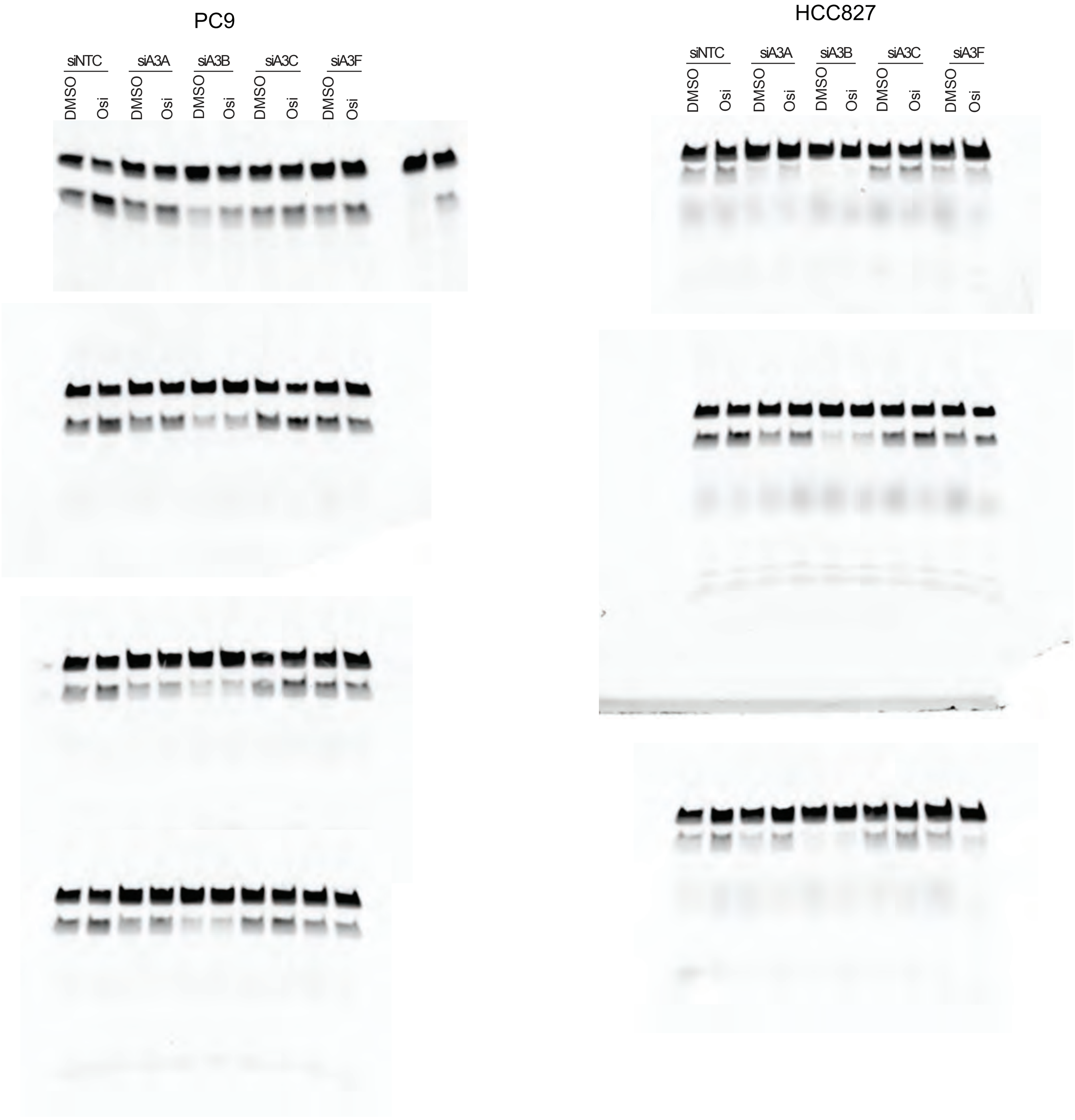
PC9
siNTC
siA3A
siA3B
siA3C
siA3F
DMSO
Osi
HCC827
siNTC
siA3A
siA3B
siA3C
siA3F
DMSO
Osi

Fig. 5e

pEGFR

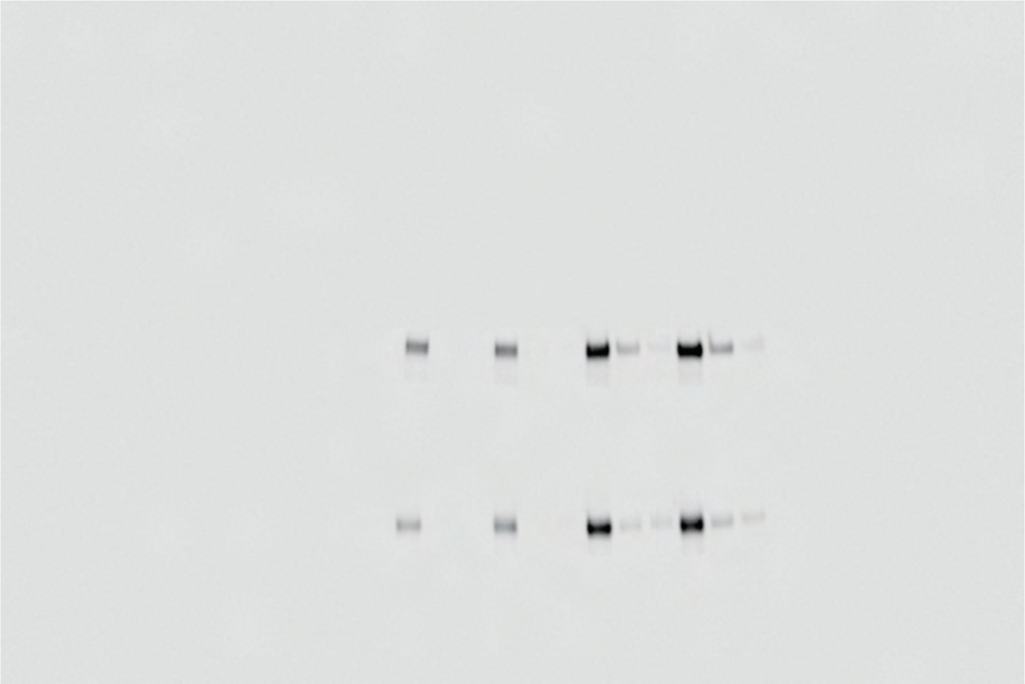

pERK

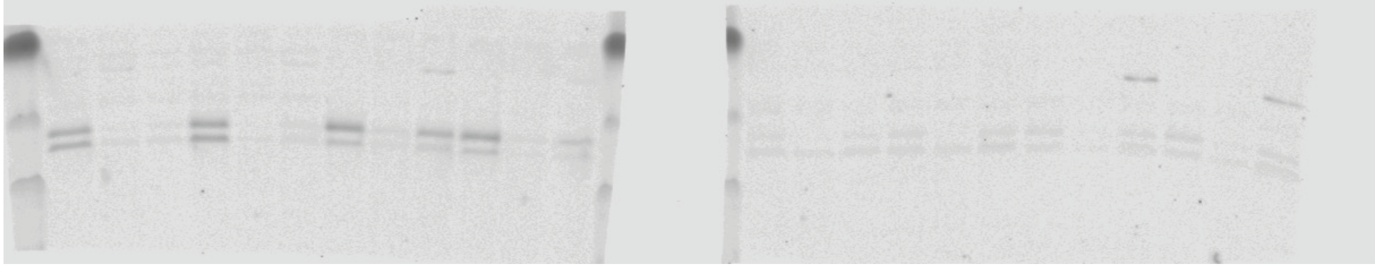

Fig. 5e

APOBEC3B

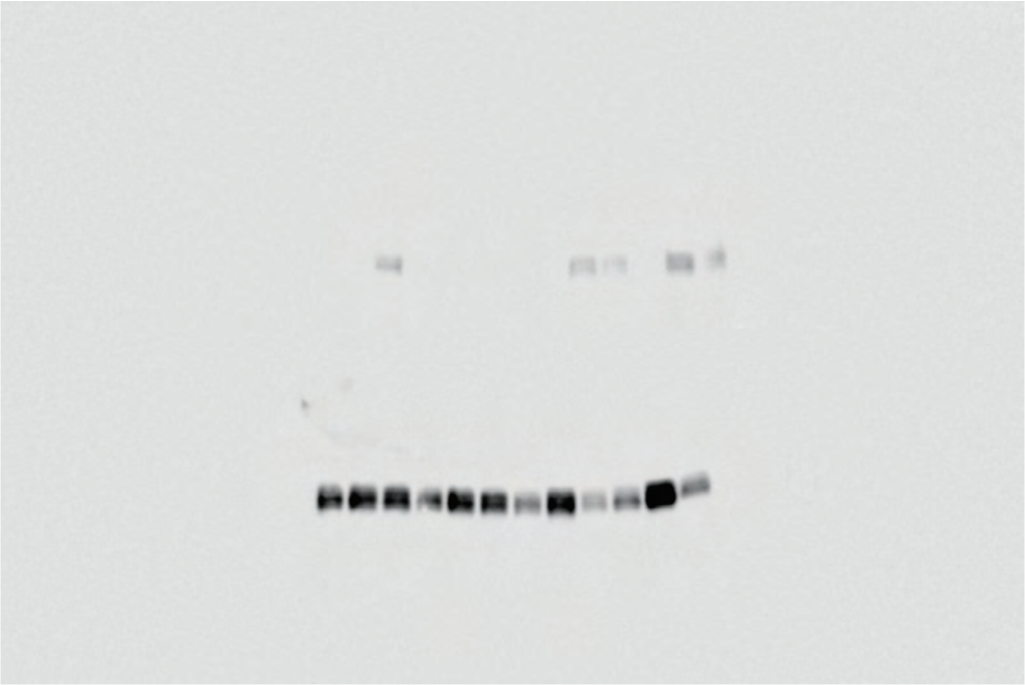

UNG

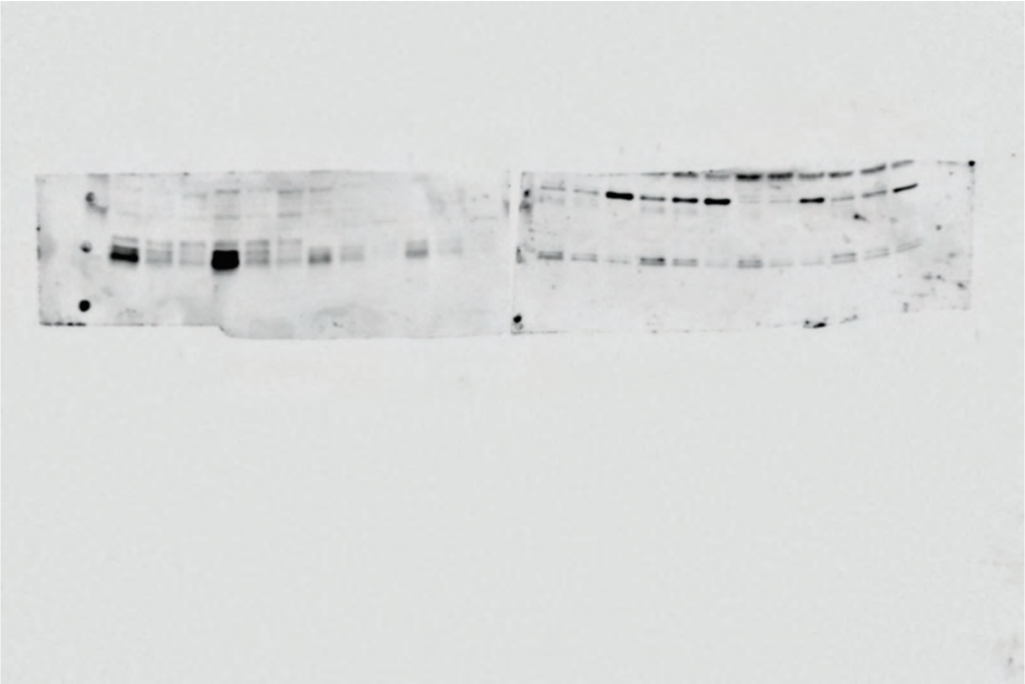

Fig. 5e

GAPDH

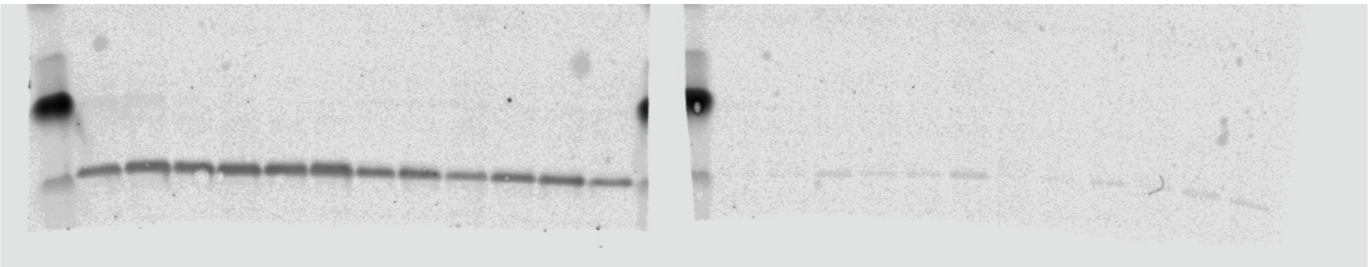

H3

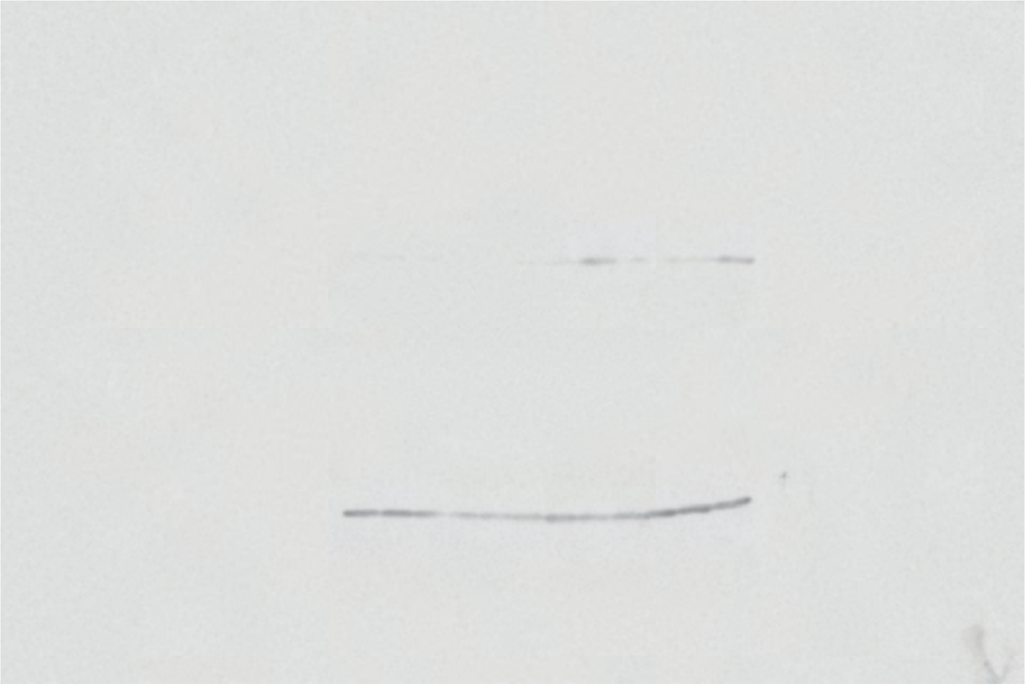

Extended Data Fig. 4a

APOBEC3B

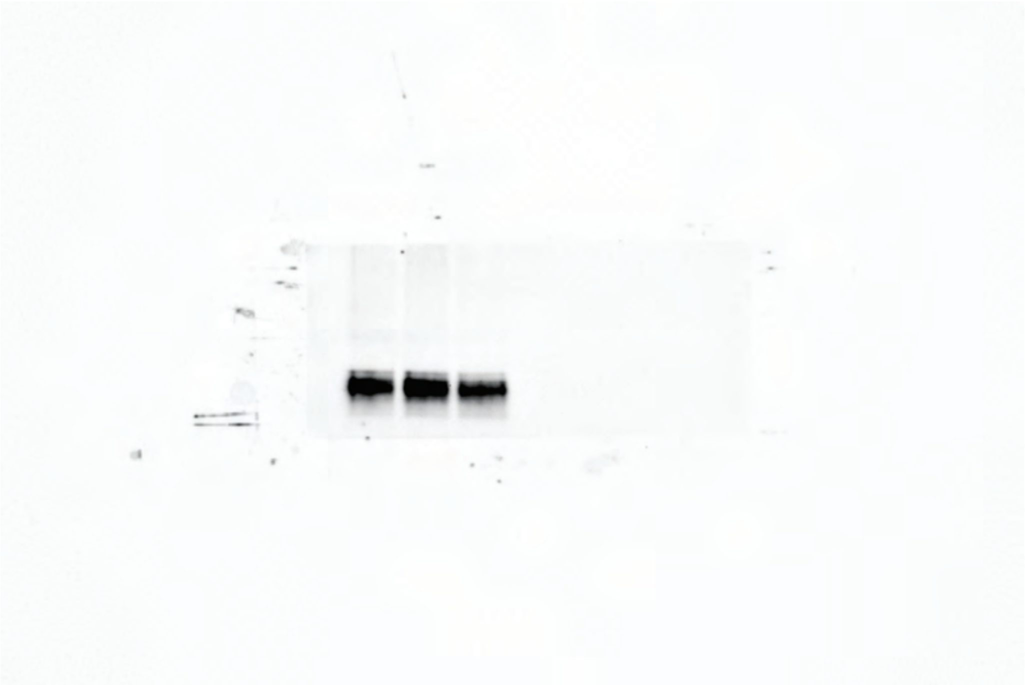

HSP90

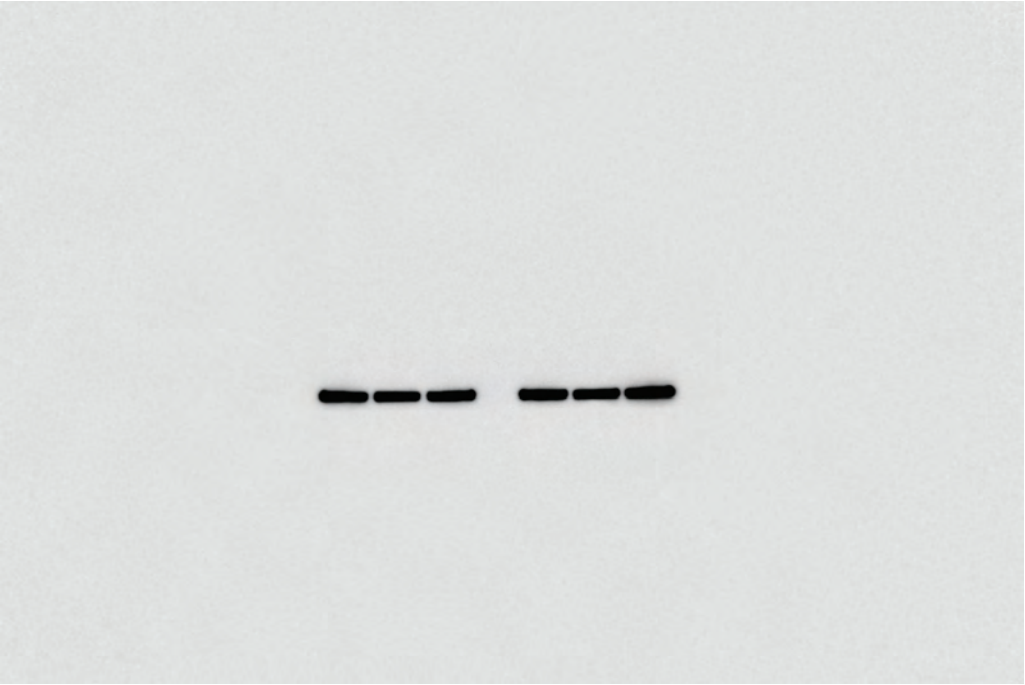

Extended Data Fig. 4c+e

APOBEC3B

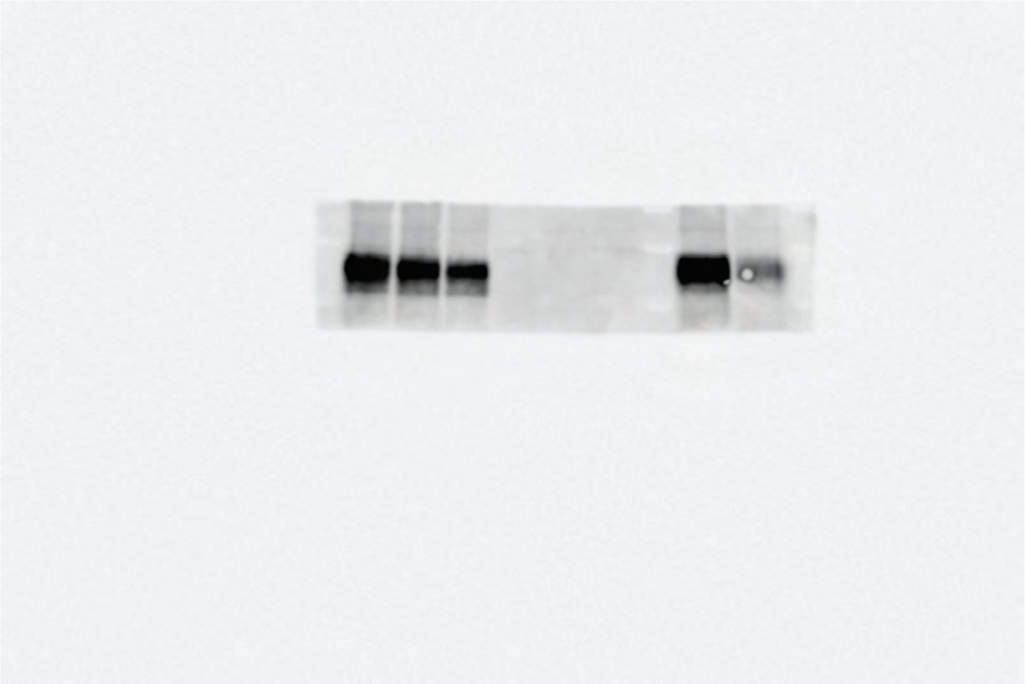

HSP90

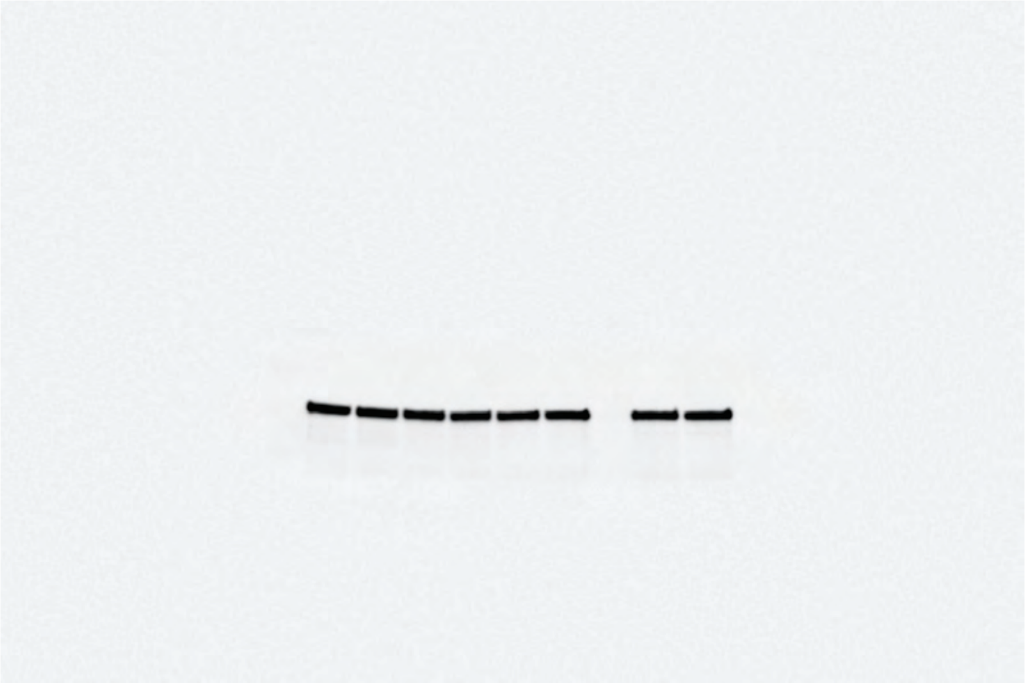

Extended Data Fig. 5a

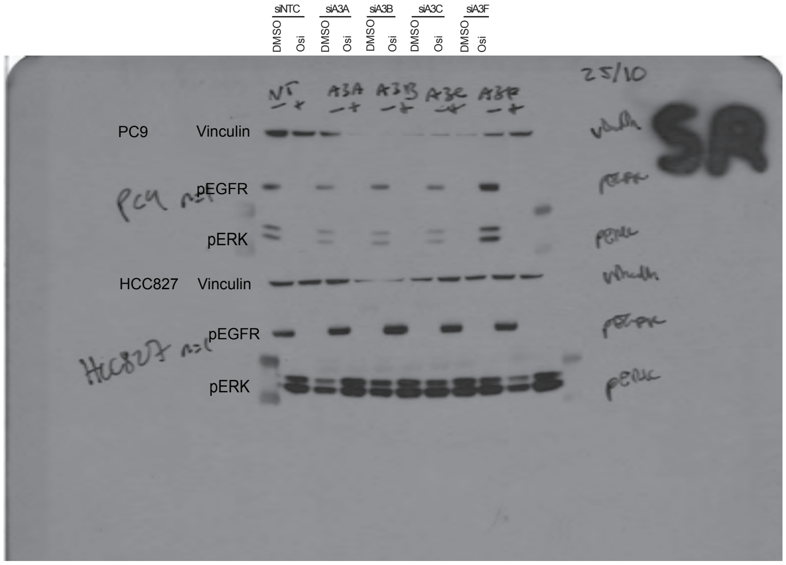

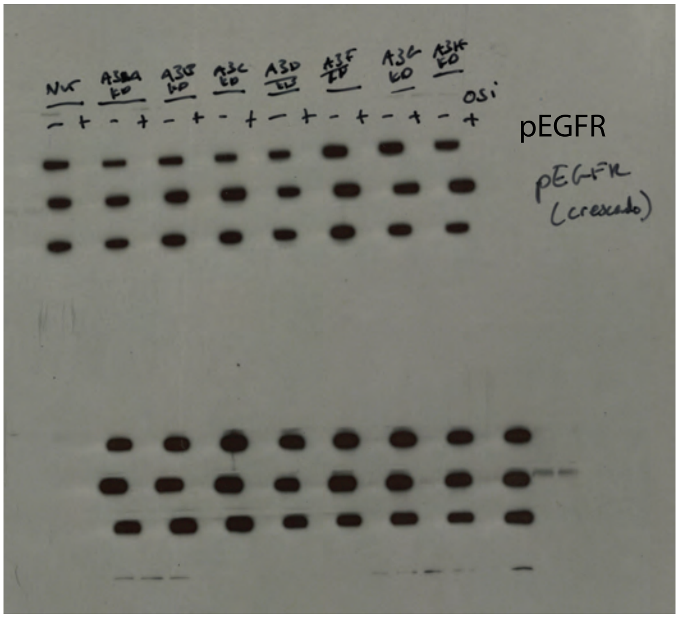

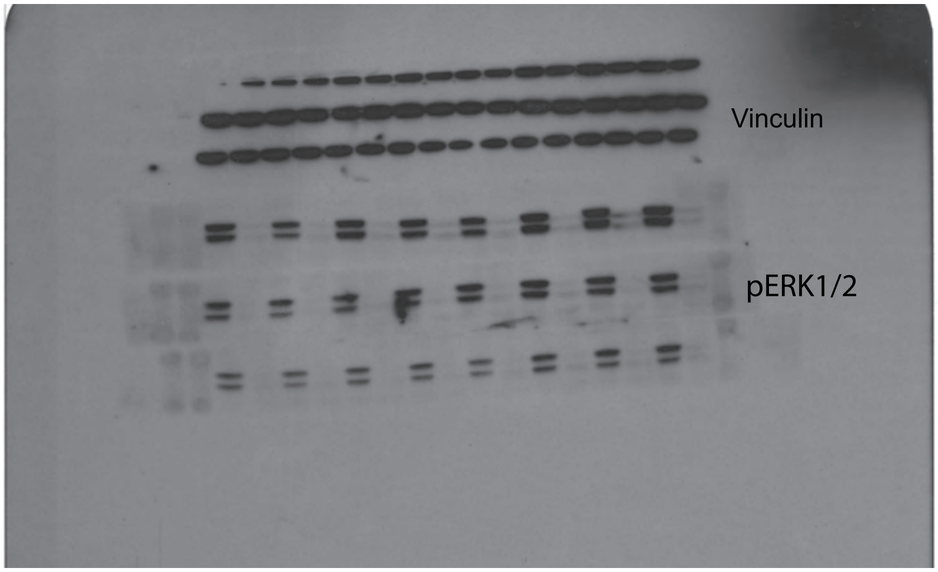

Ext. Data. Fig. 6c

pEGFR

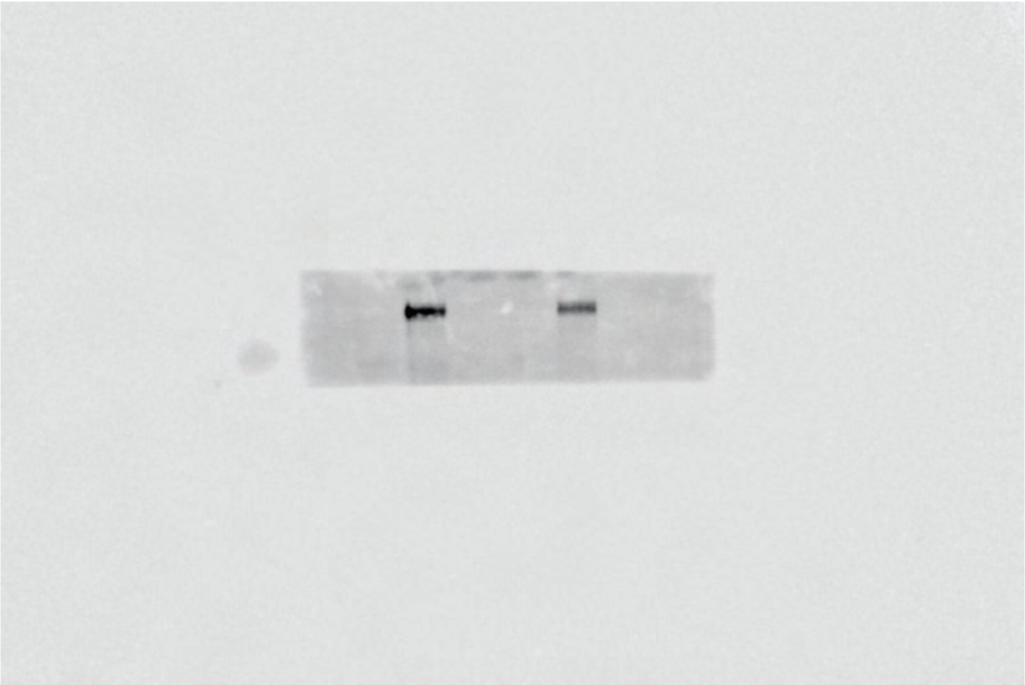

APOBEC3B

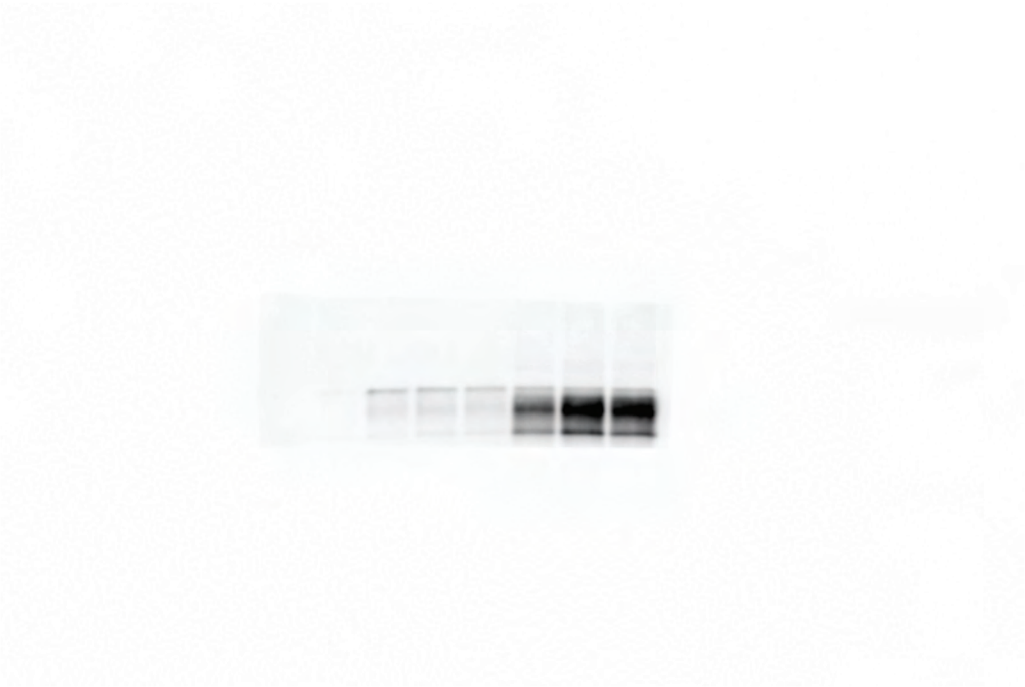

Ext. Data. Fig. 6c

GAPDH

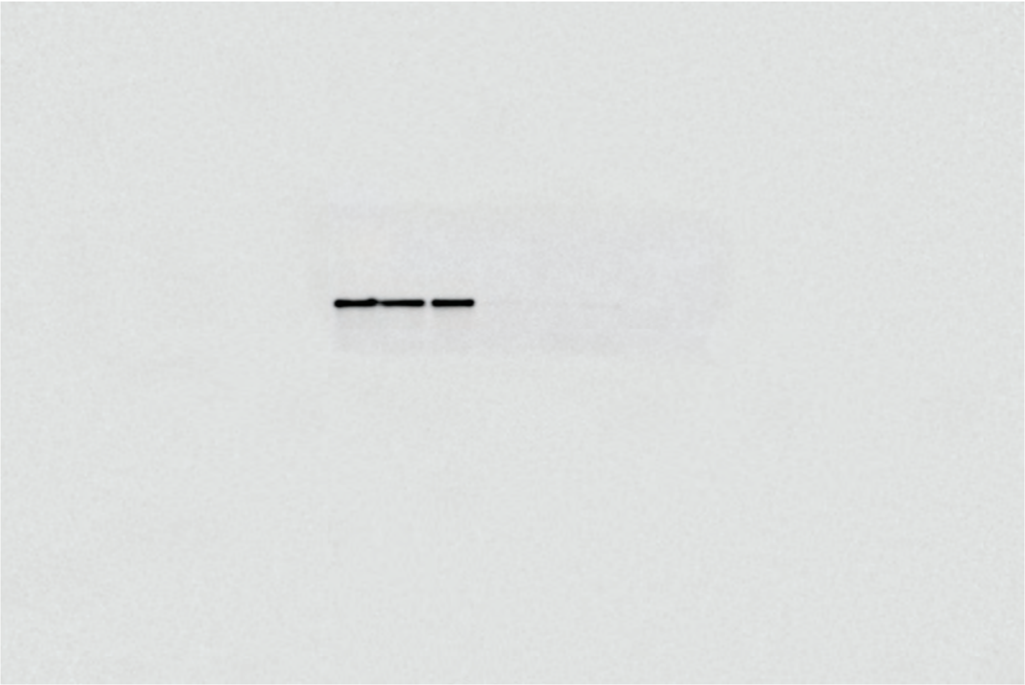

H3

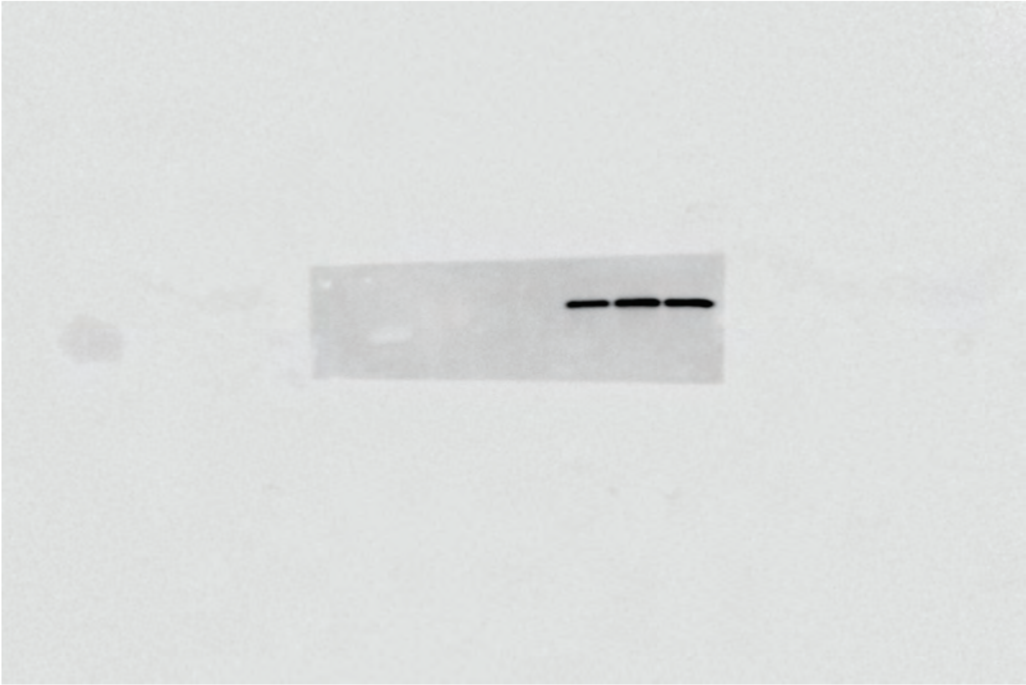

Ext. Data. Fig. 6f

APOBEC3B

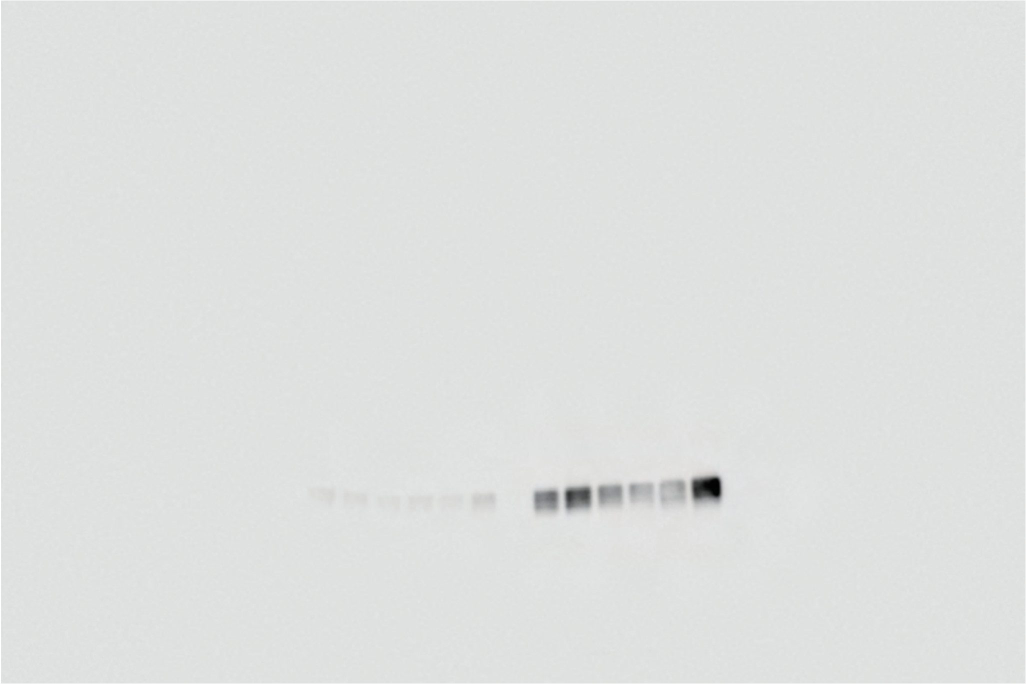

pERK

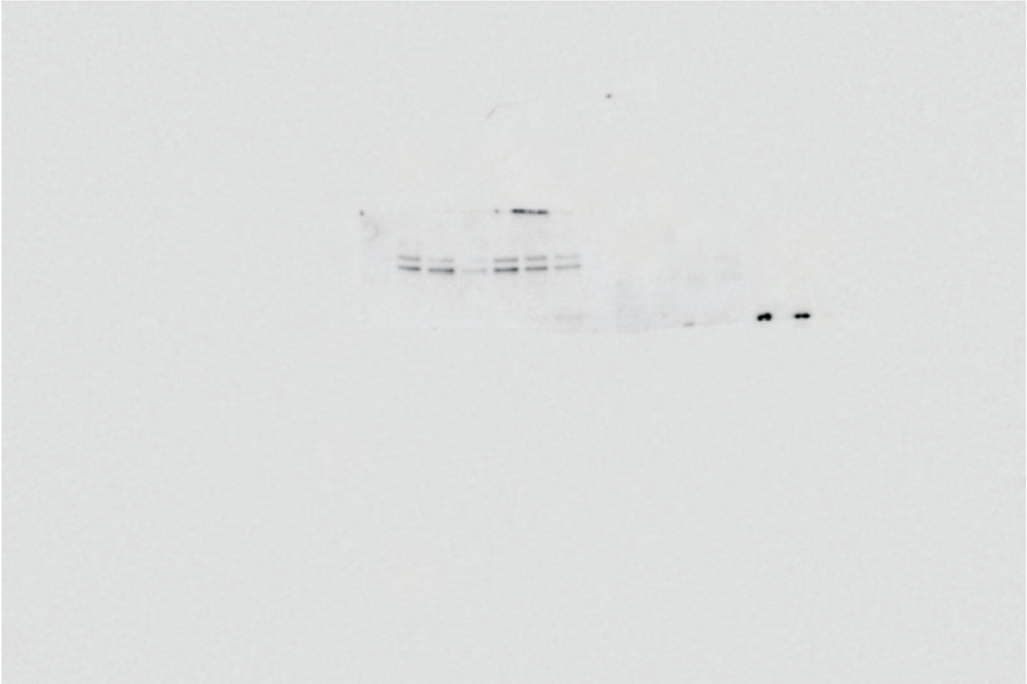

Extended Data. Fig. 6f

UNG

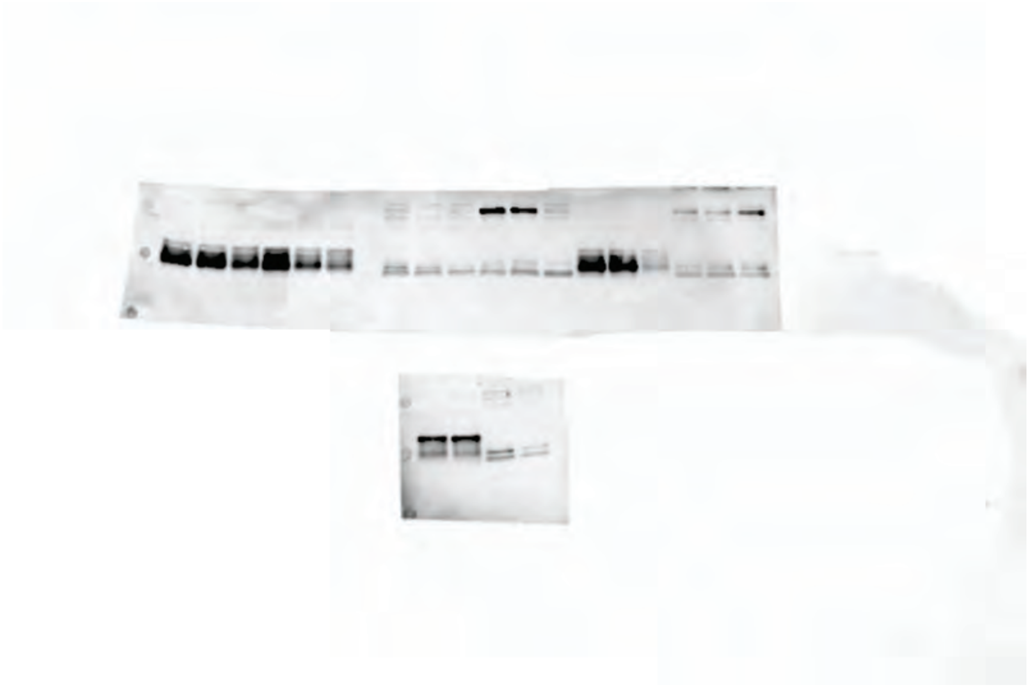

Extended Data. Fig. 6f

H3

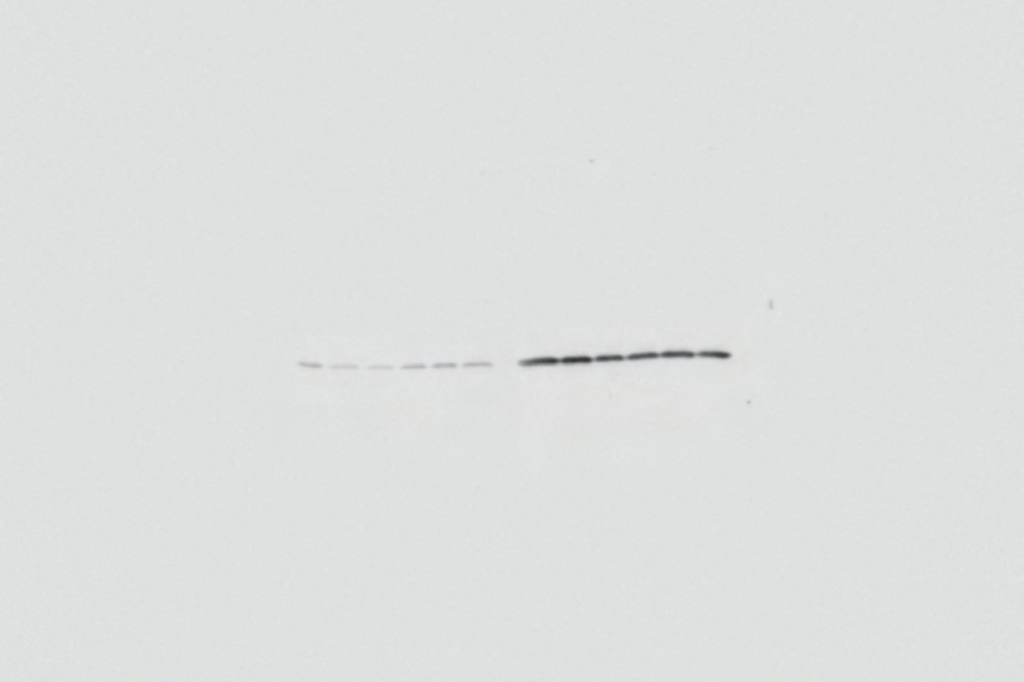

TUBB

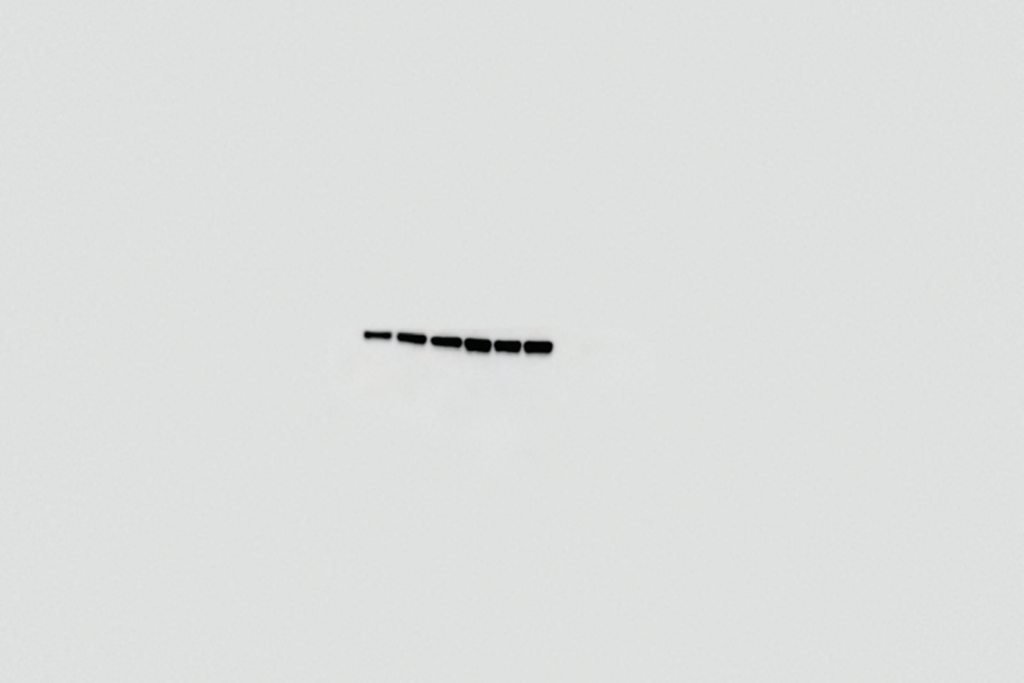

Extended Data Fig. 6i

APOBEC3B

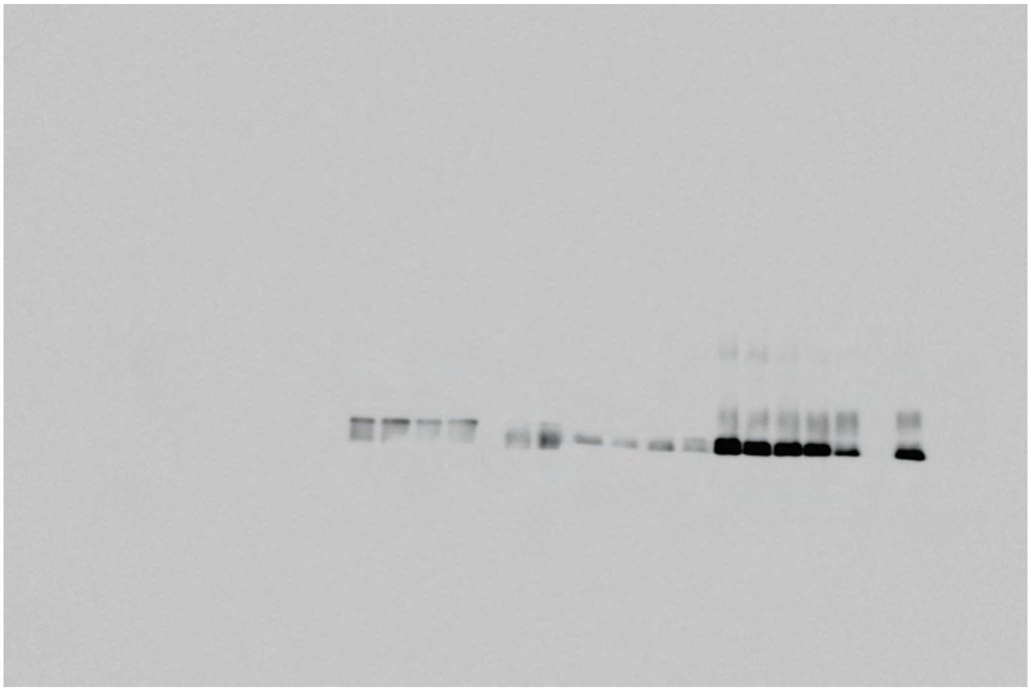

UNG

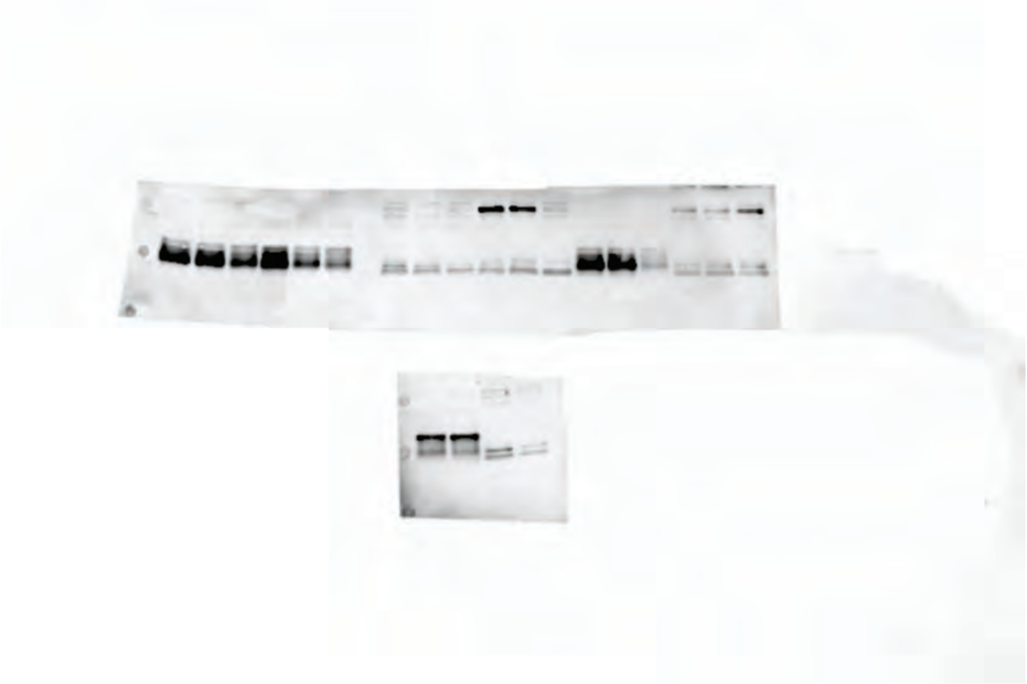

Extended Data Fig. 6i

H3

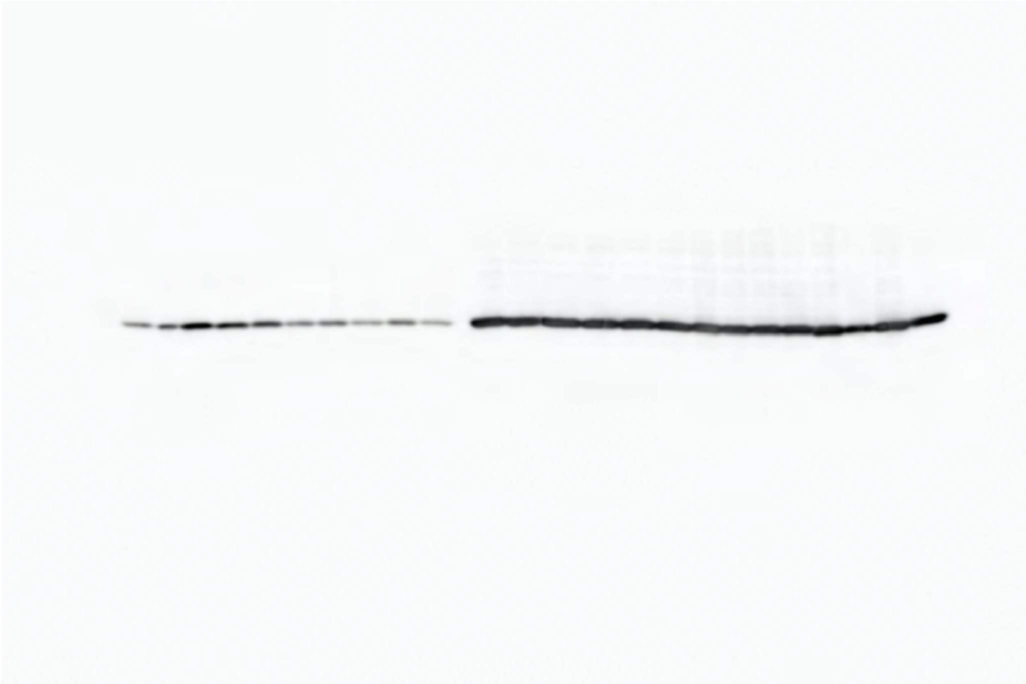

HSP90

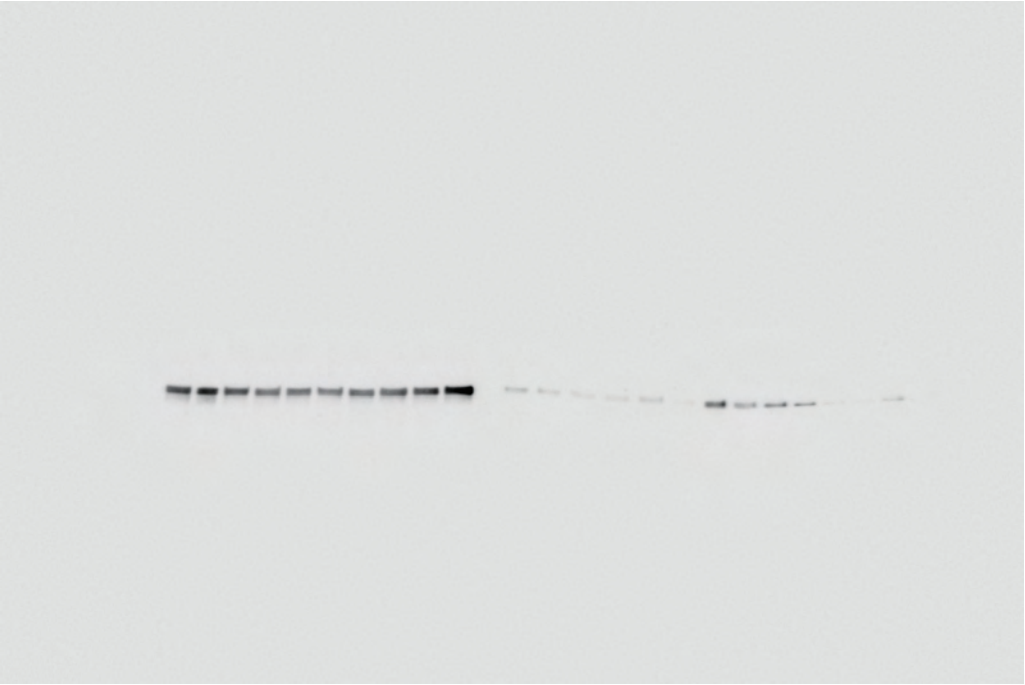

Extended Data Fig. 6j

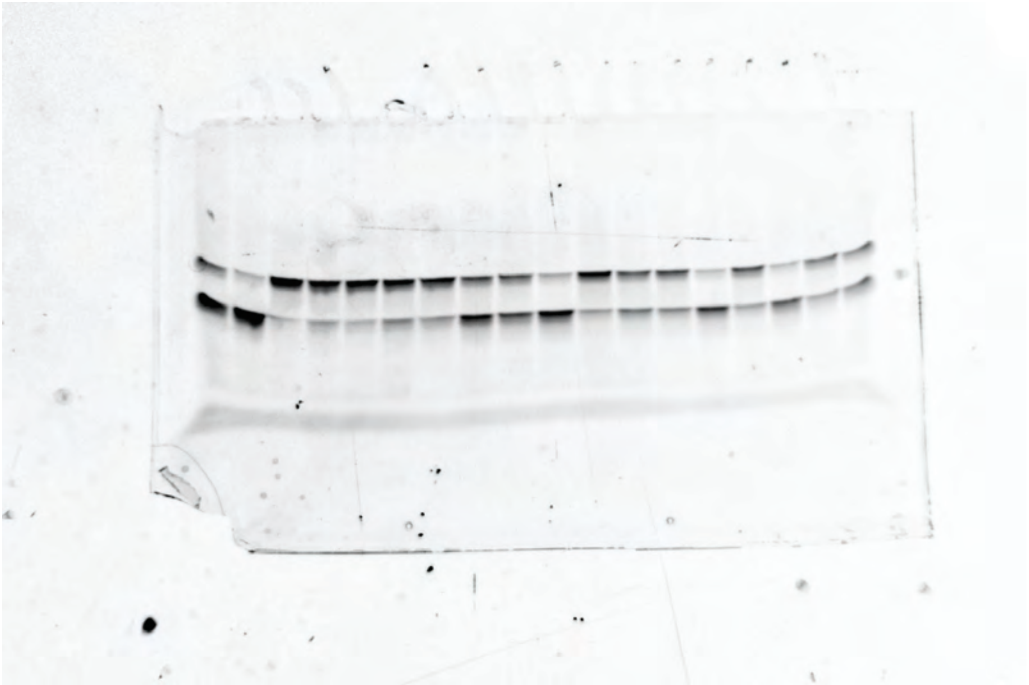

Extended Data Fig. 6k

APOBEC3B

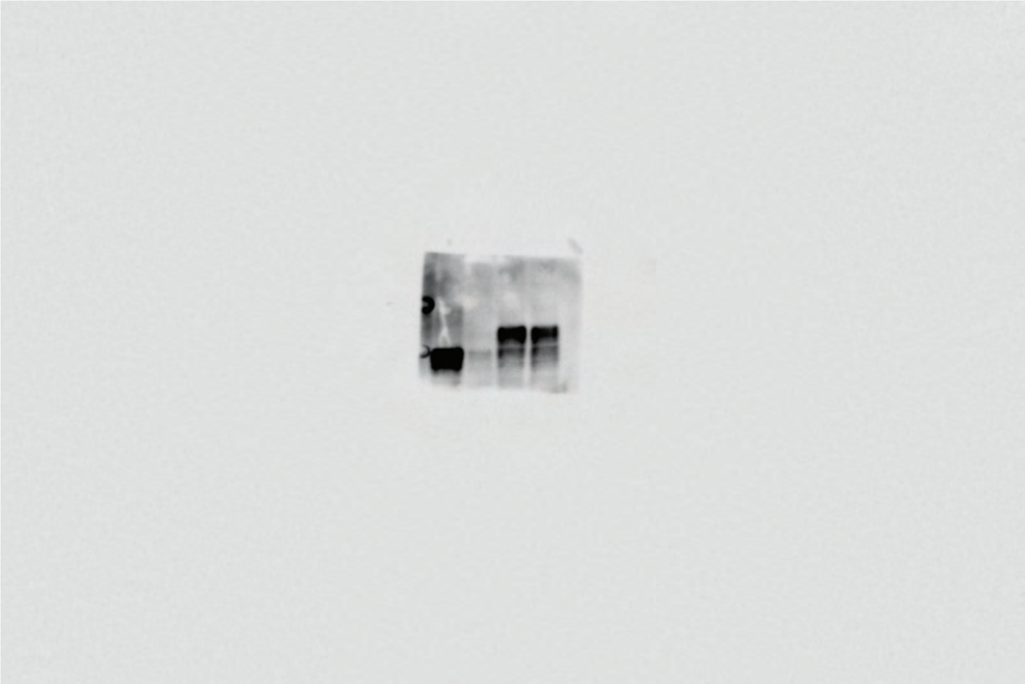

H3

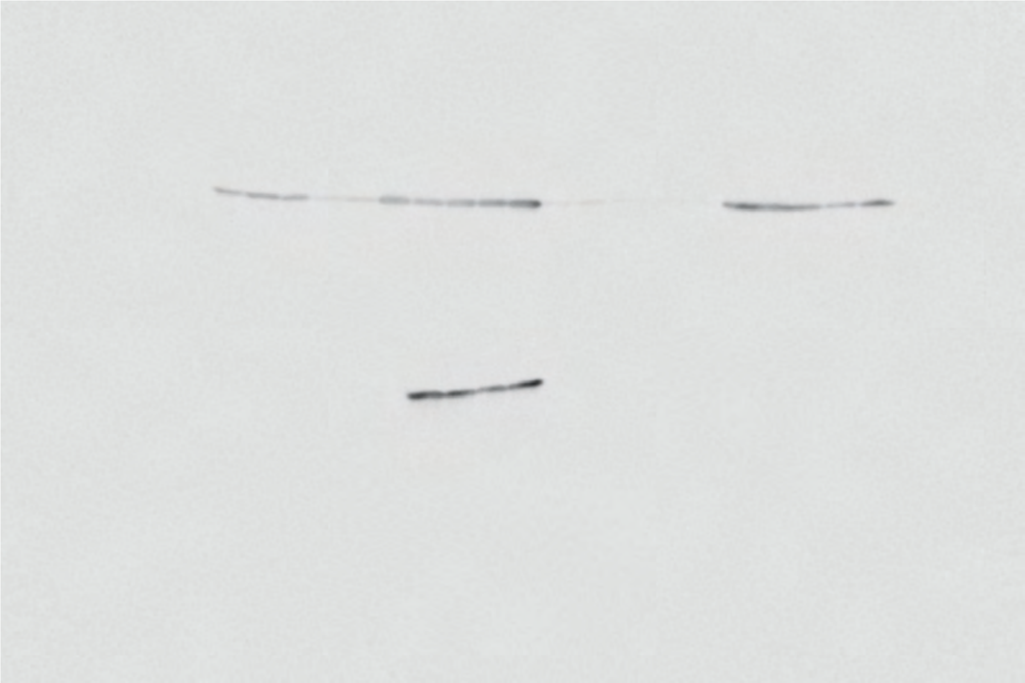

Extended Data Fig. 6l

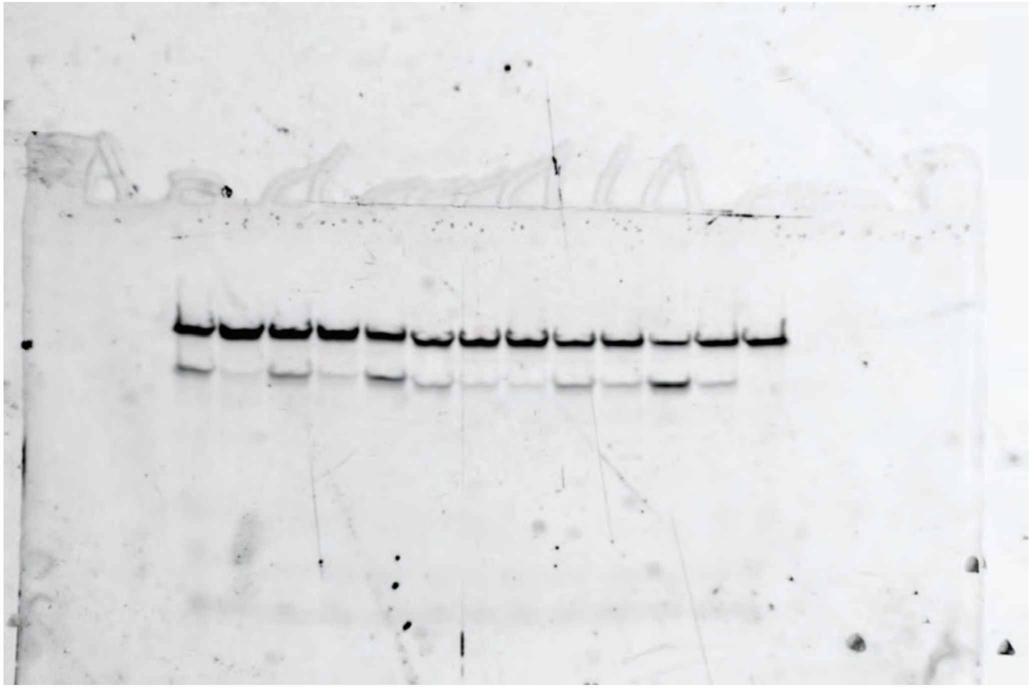

Extended Data Fig. 7a

APOBEC3B

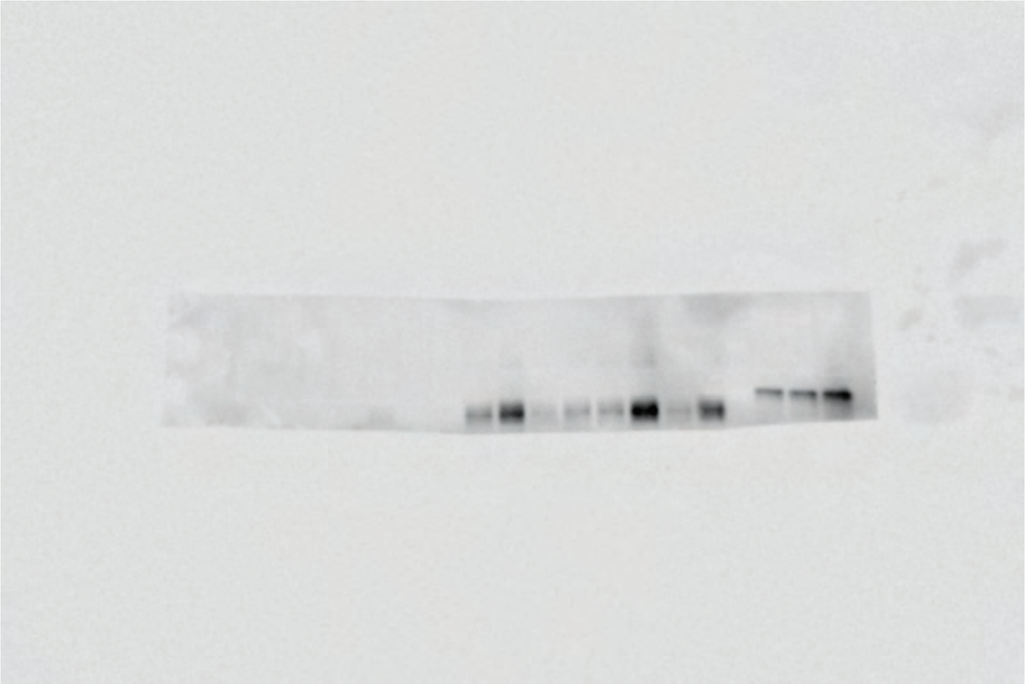

UNG

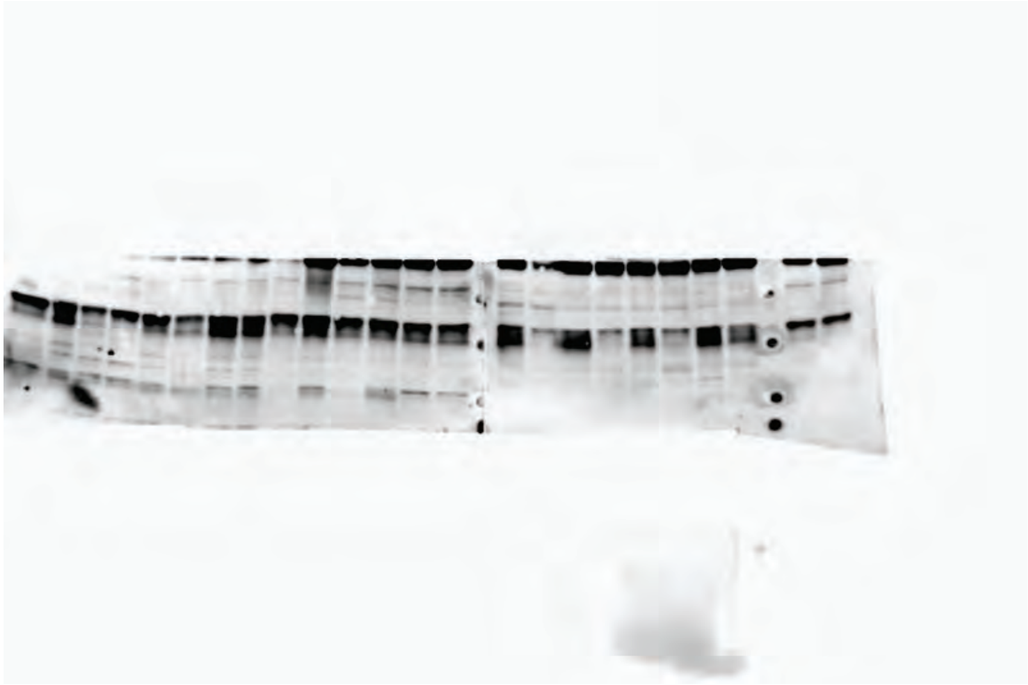

Extended Data Fig. 7*a*

pEGFR

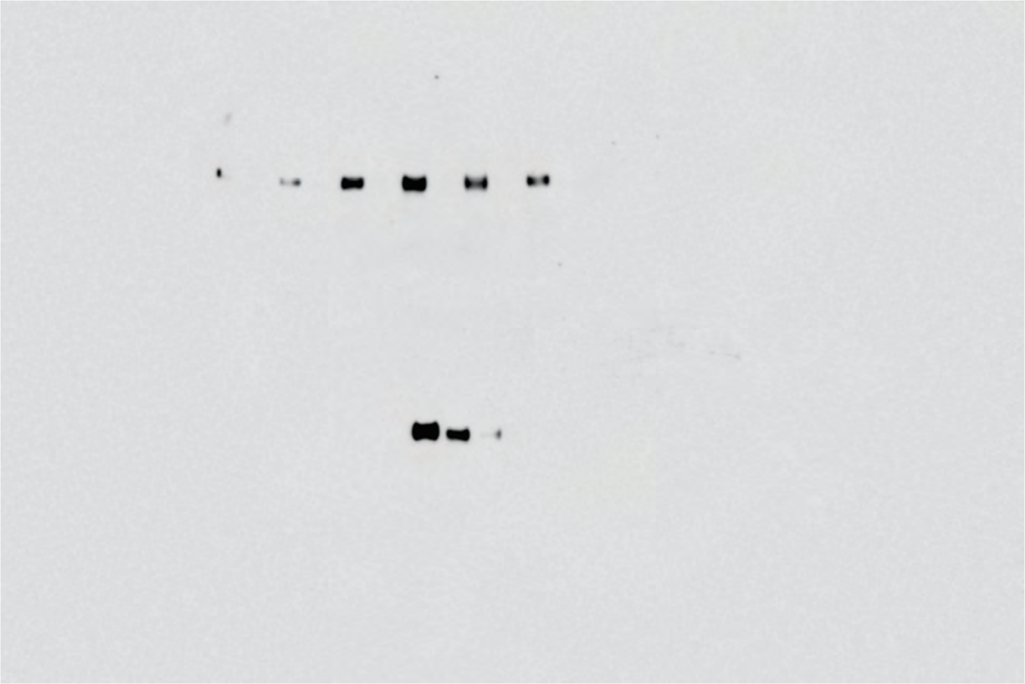

EGFR

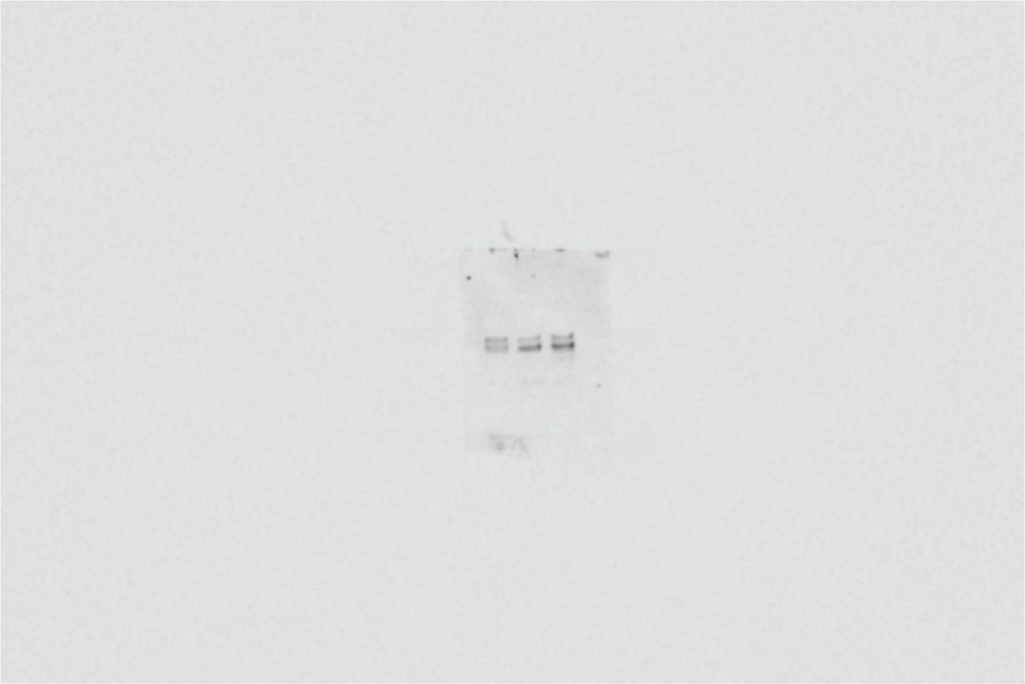

Extended Data Fig. 7a

TUBB

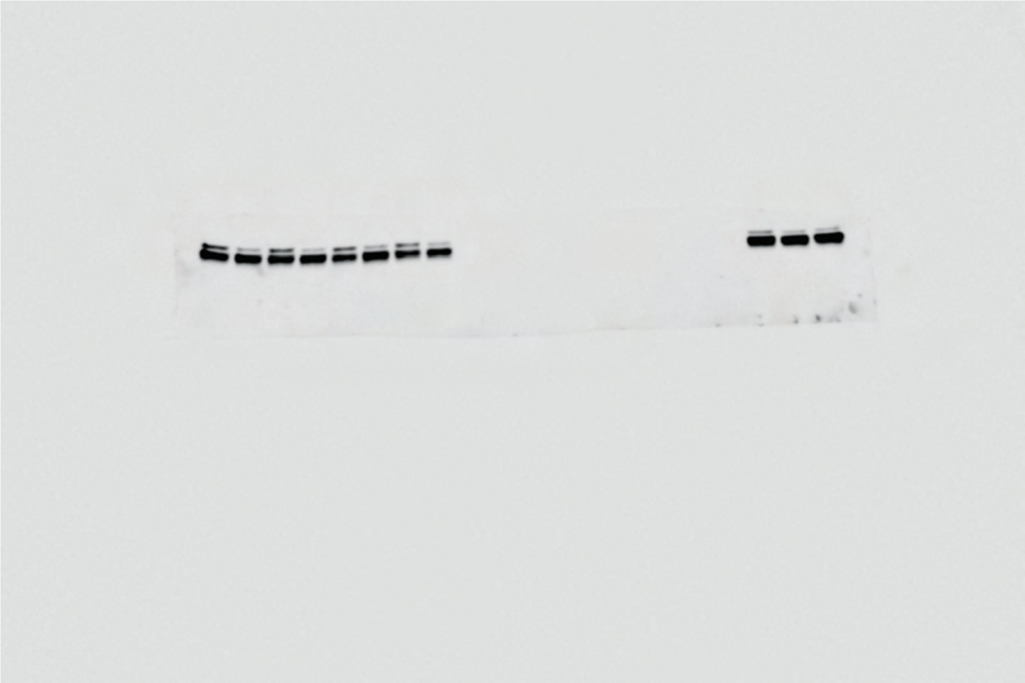

Extended Data Fig. 7b

APOBEC3B

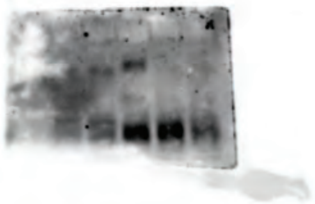

UNG

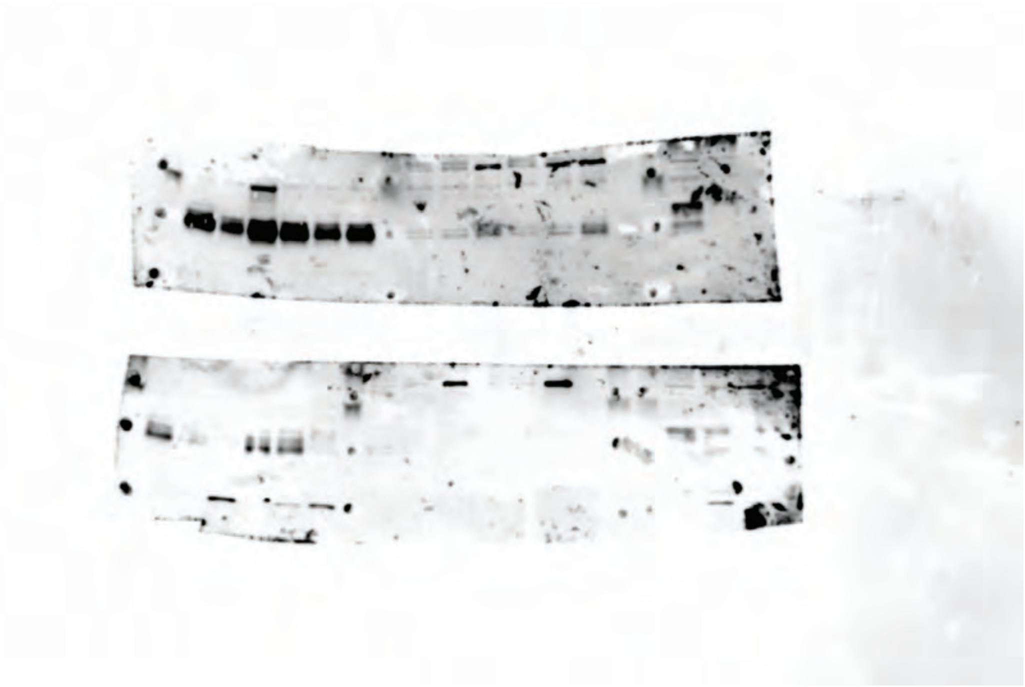

Extended Data Fig. 7b

pEGFR

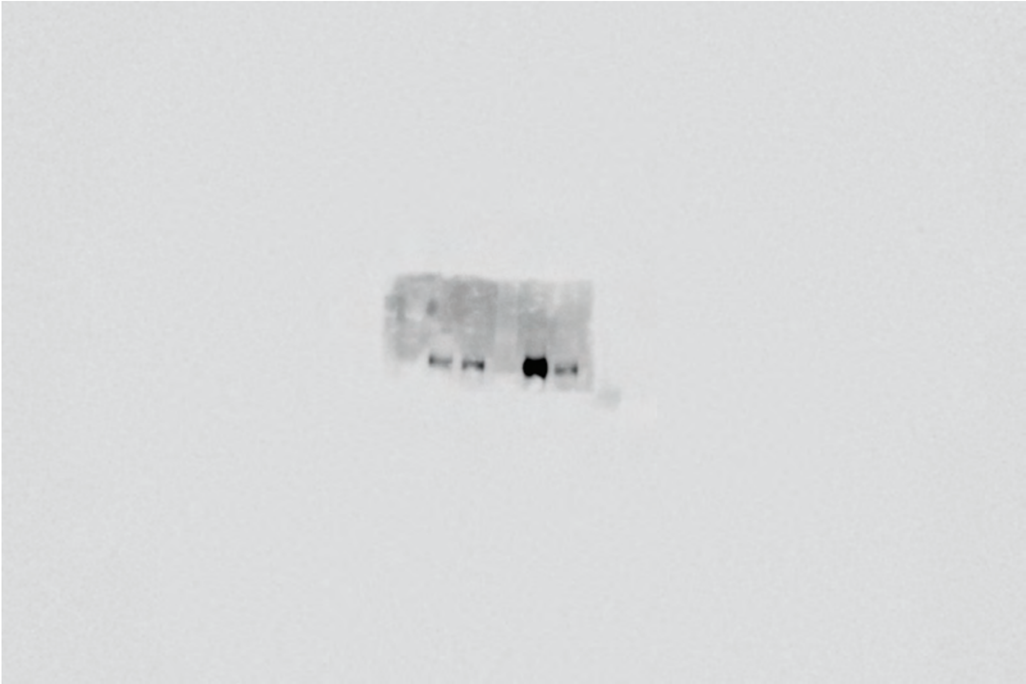

HSP90

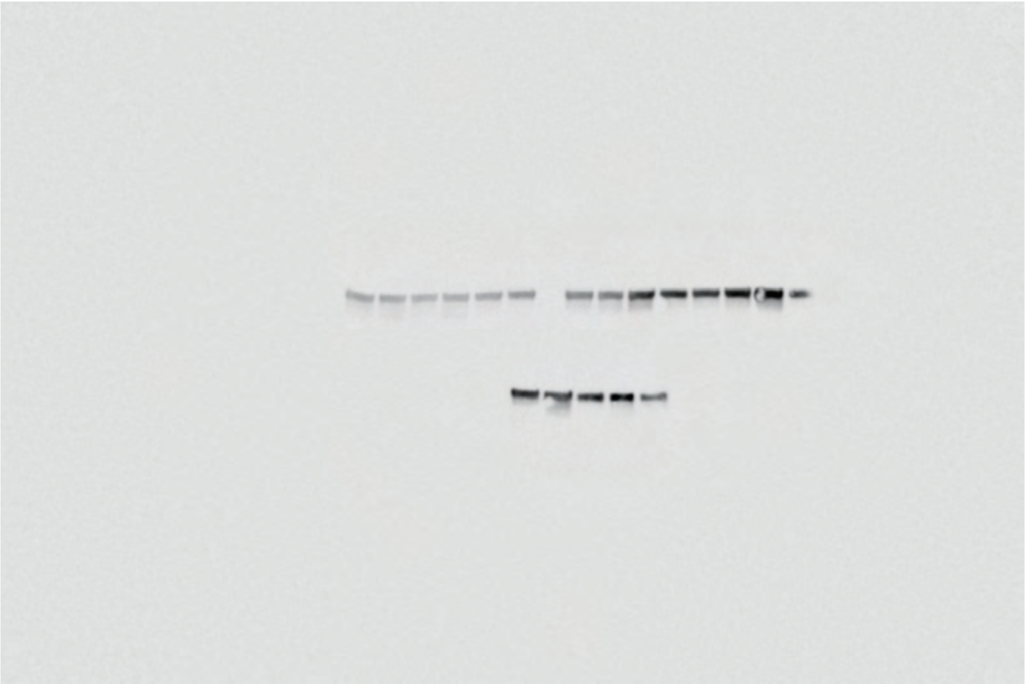

Extended Data Fig. 7b

APOBEC3B

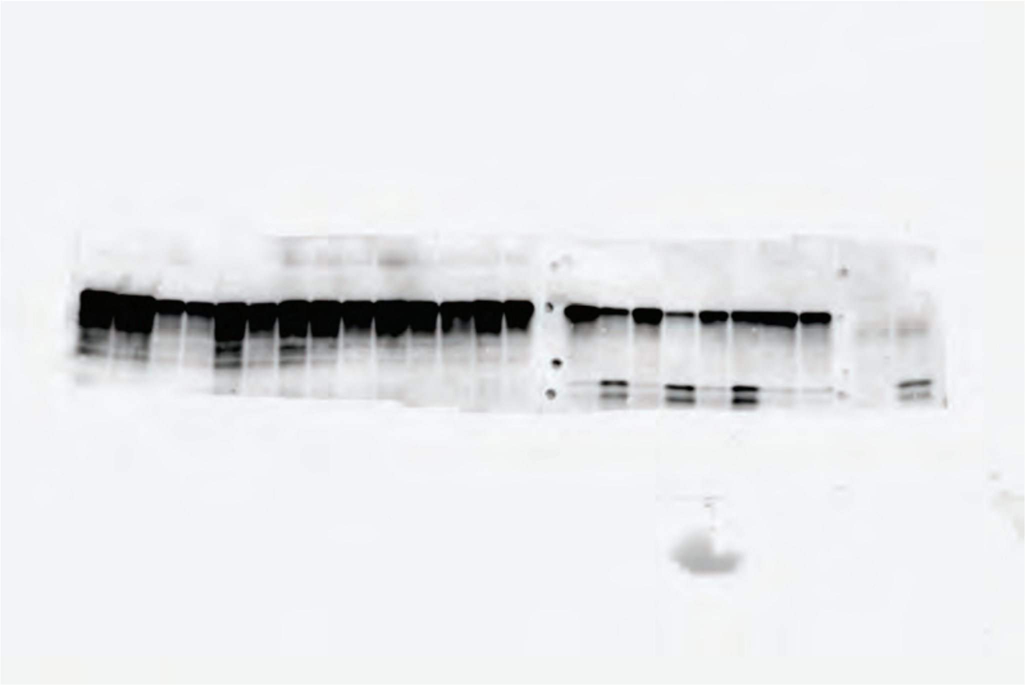

GAPDH

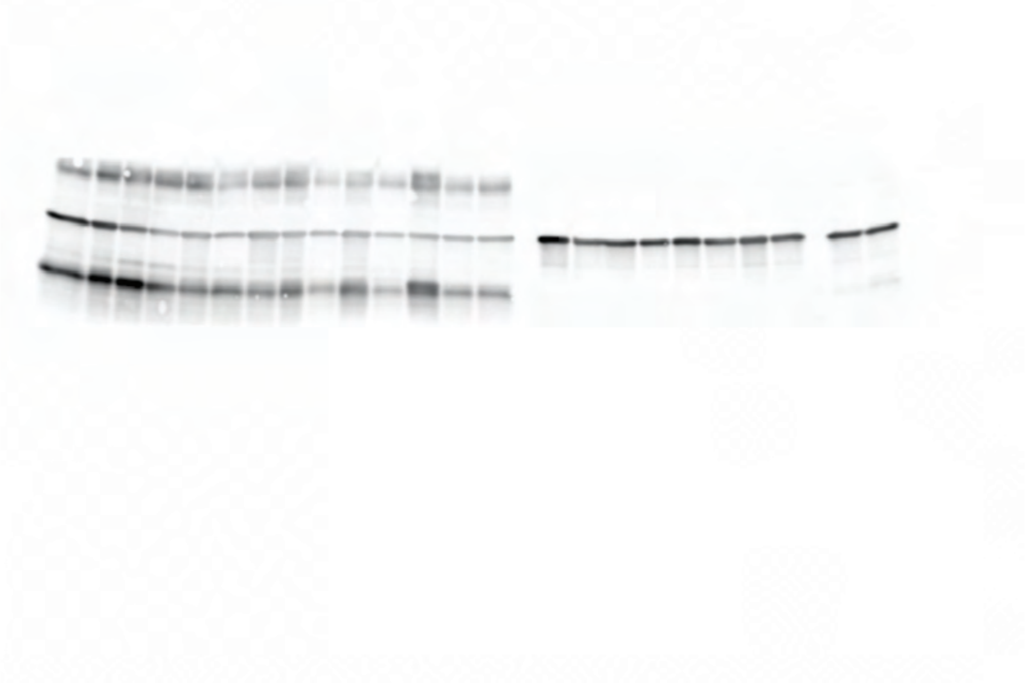

Extended Data Fig. 8b

APOBEC3B

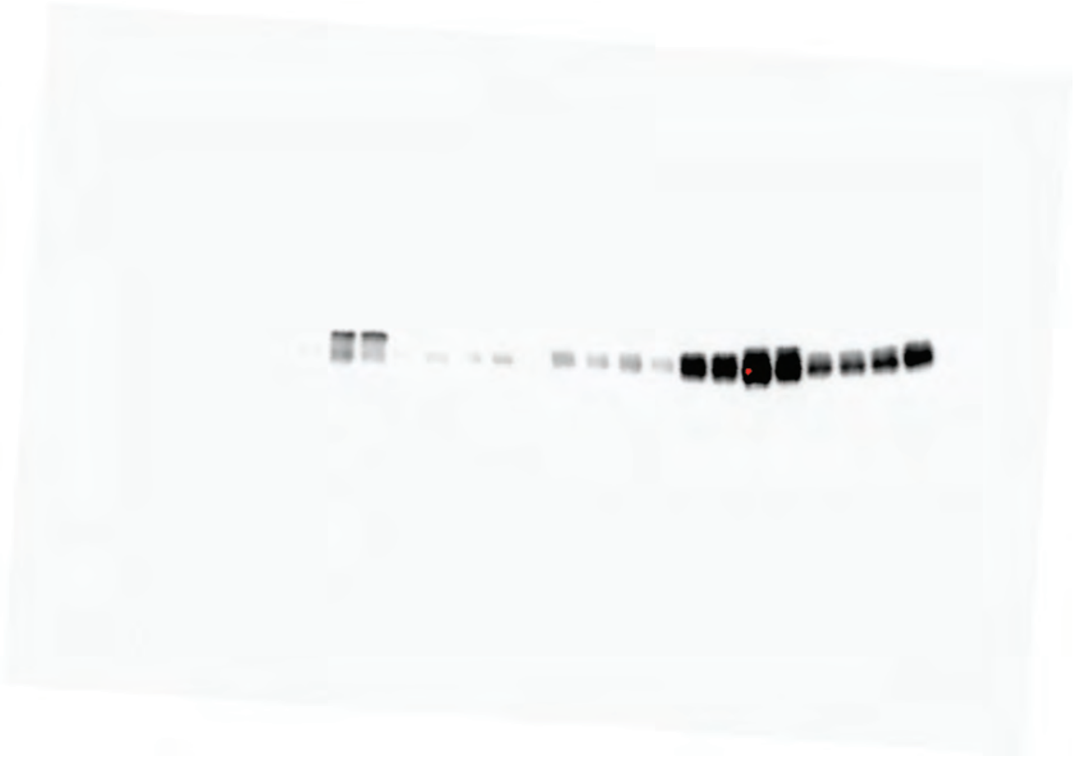

Extended Data Fig. 8b

RelA

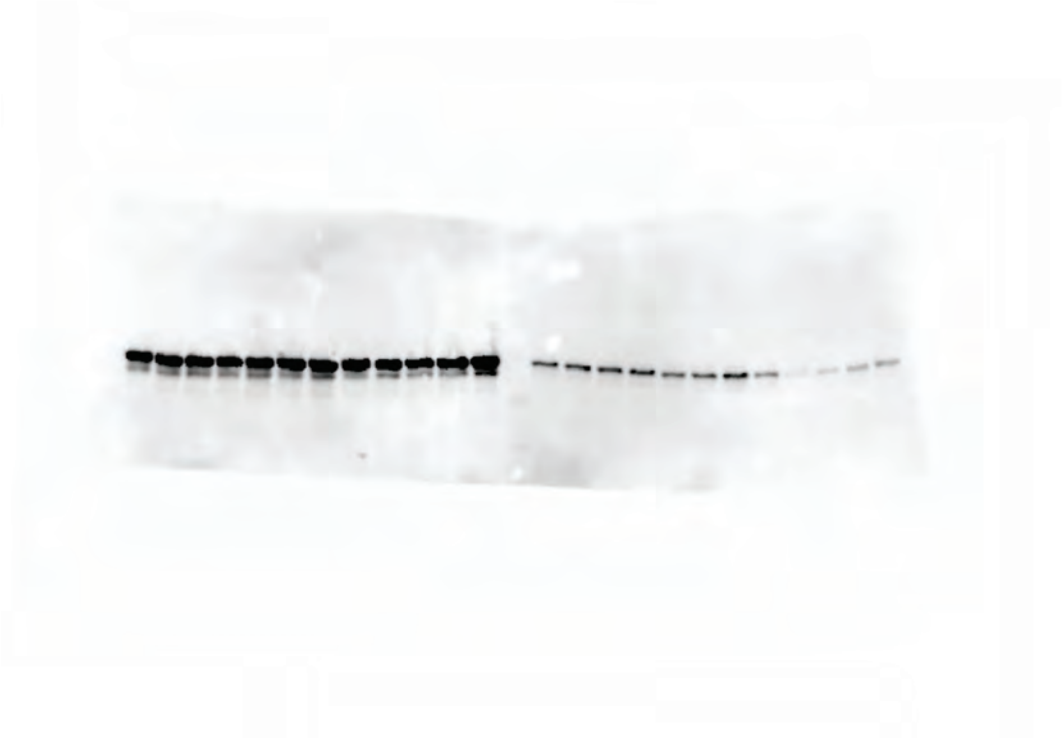

RelB

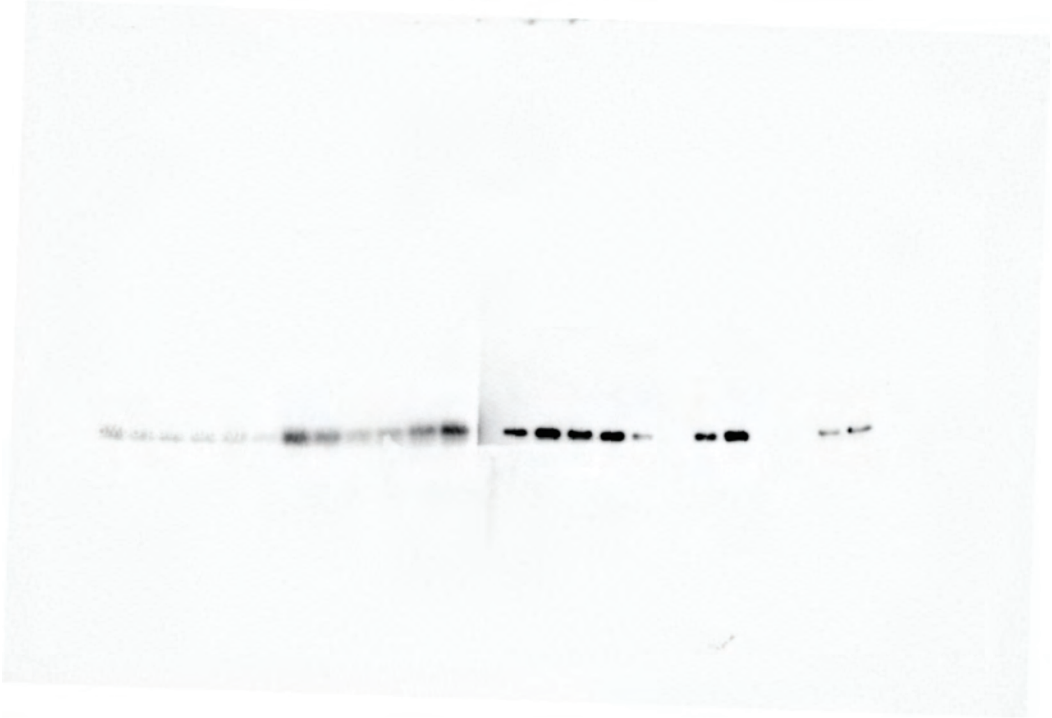

Extended Data Fig. 8b

H3

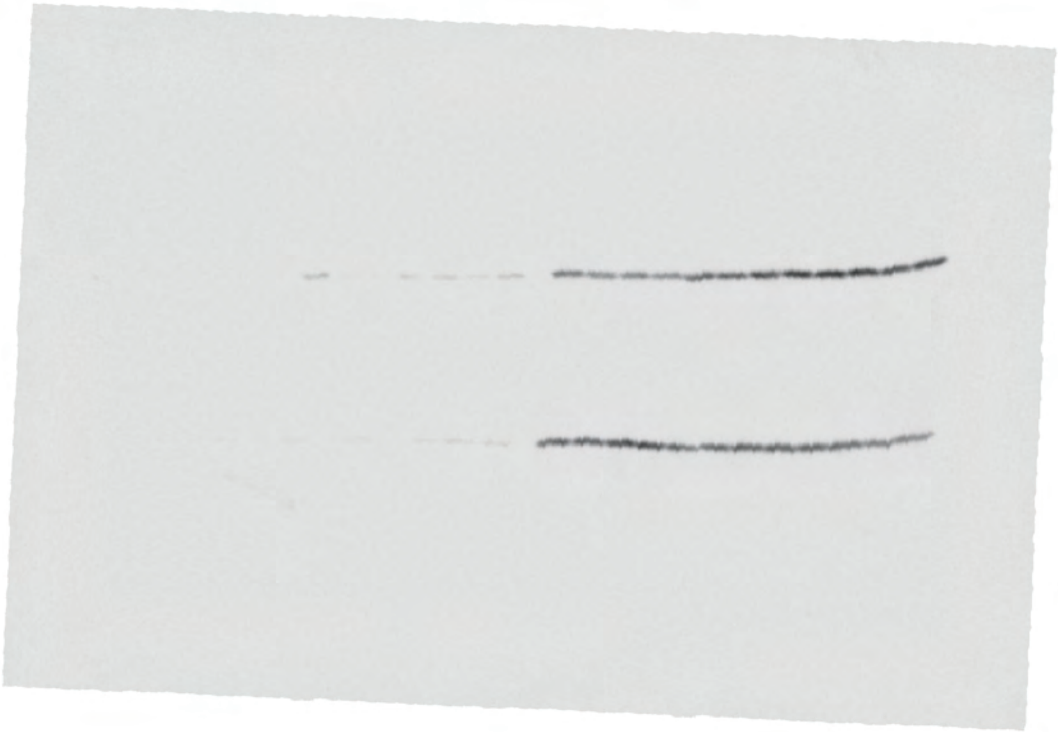

HSP90

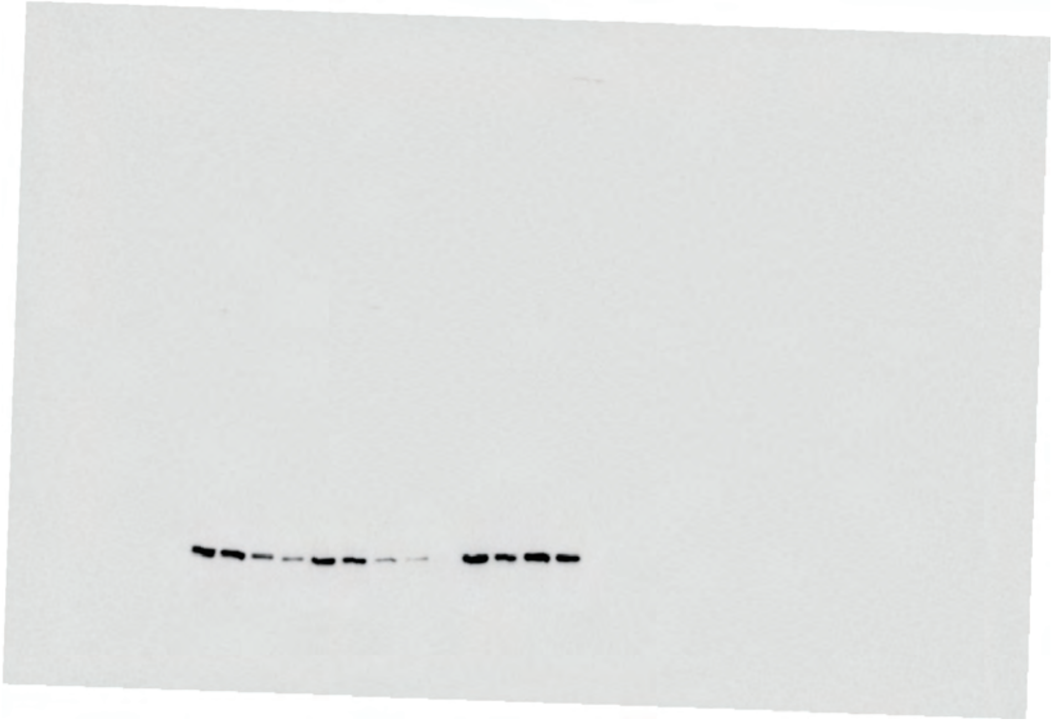

Extended Data Fig. 8f

pEGFR

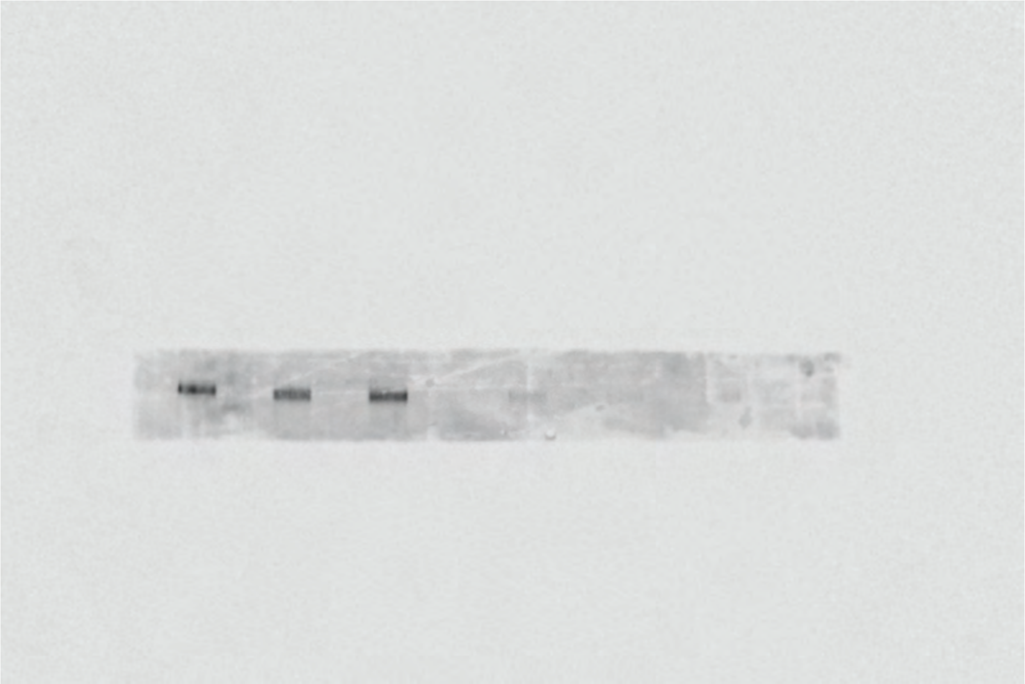

APOBEC3B

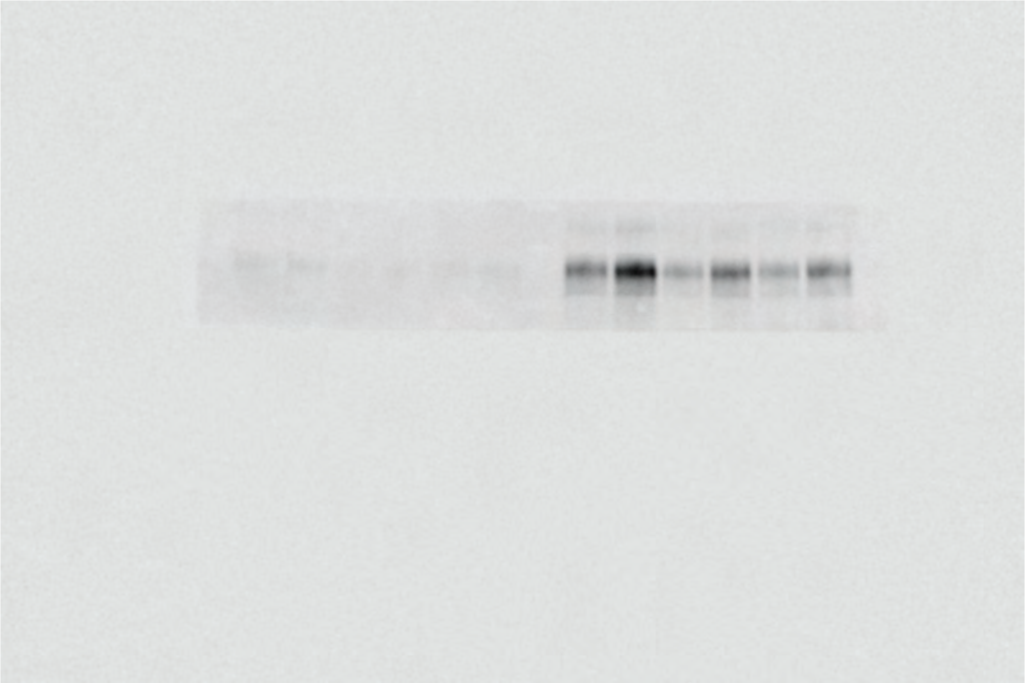

Extended Data Fig. 8f

RelA CYT

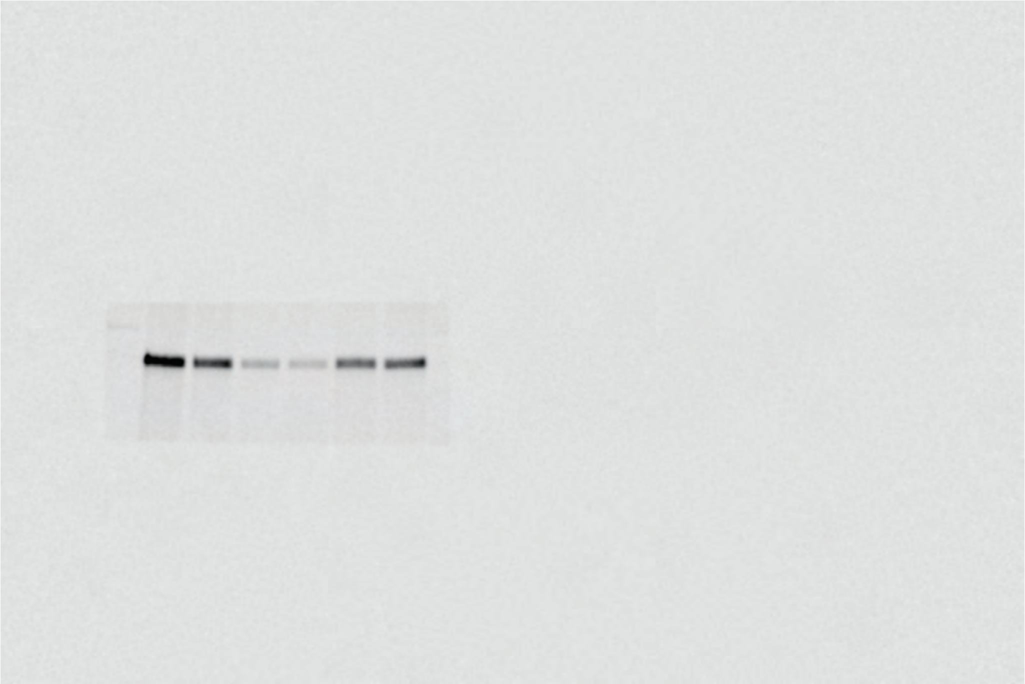

RelA NUC

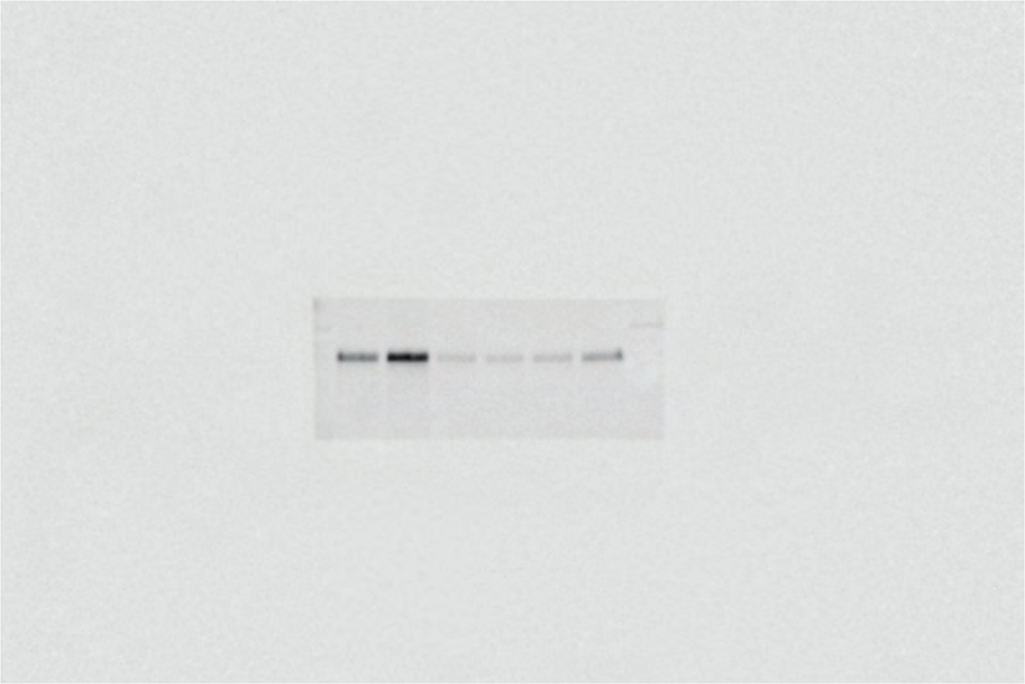

Extended Data Fig. 8f

RelB CYT

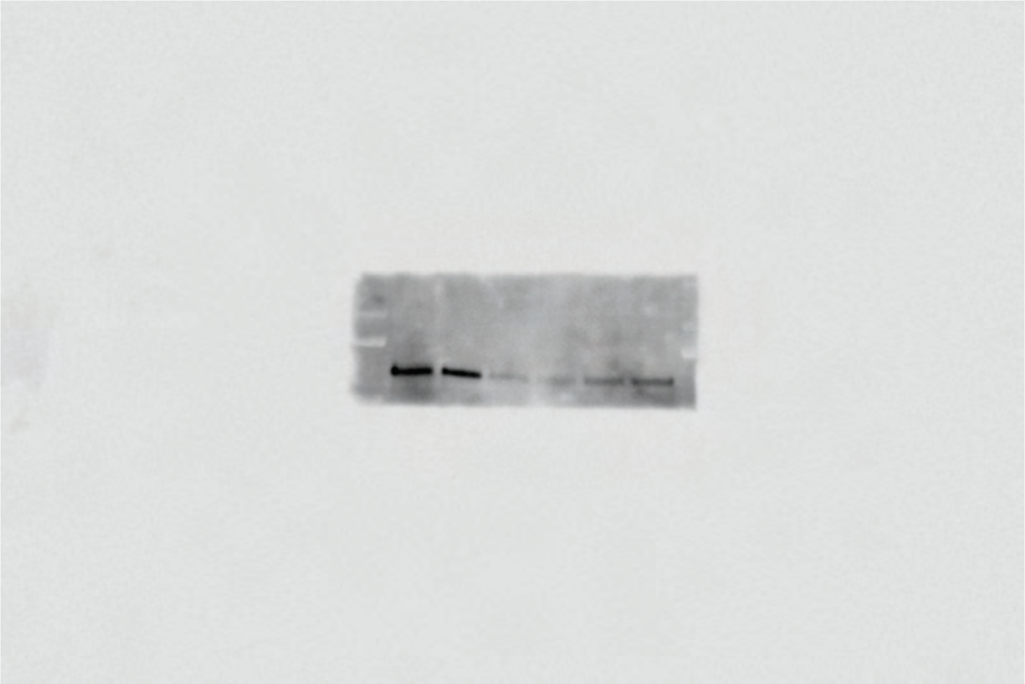

RelB NUC

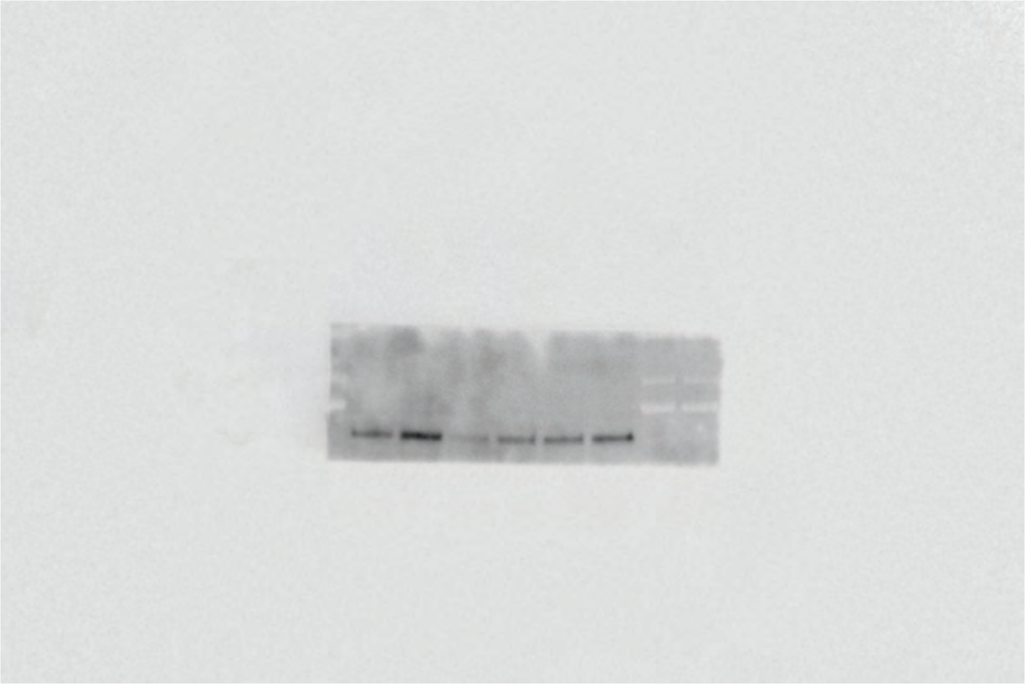

Extended Data Fig. 8f

GAPDH

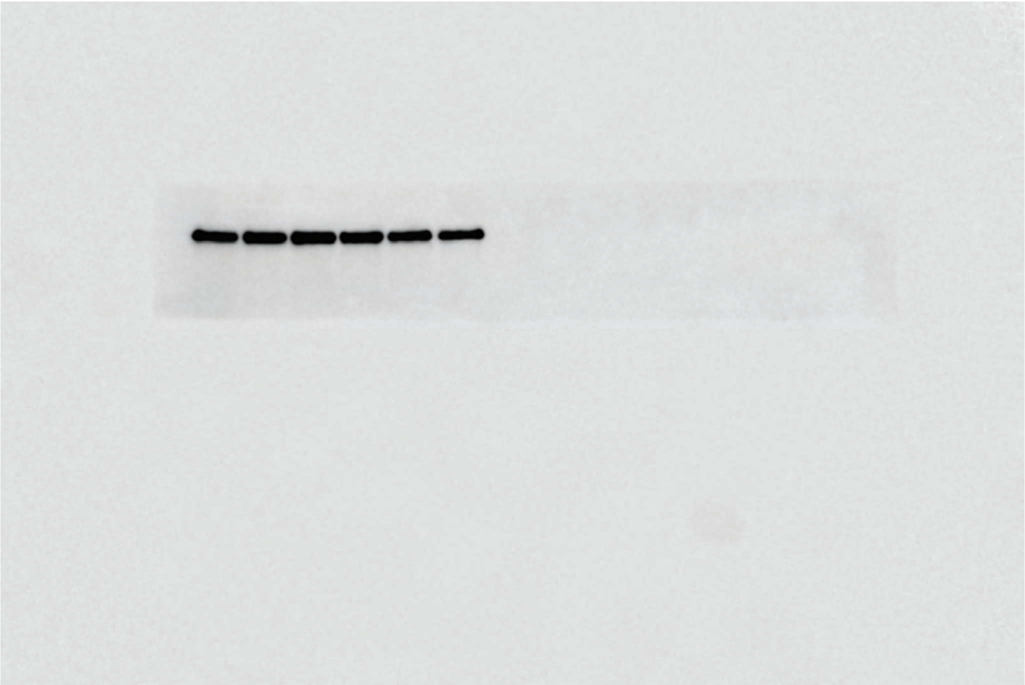

H3

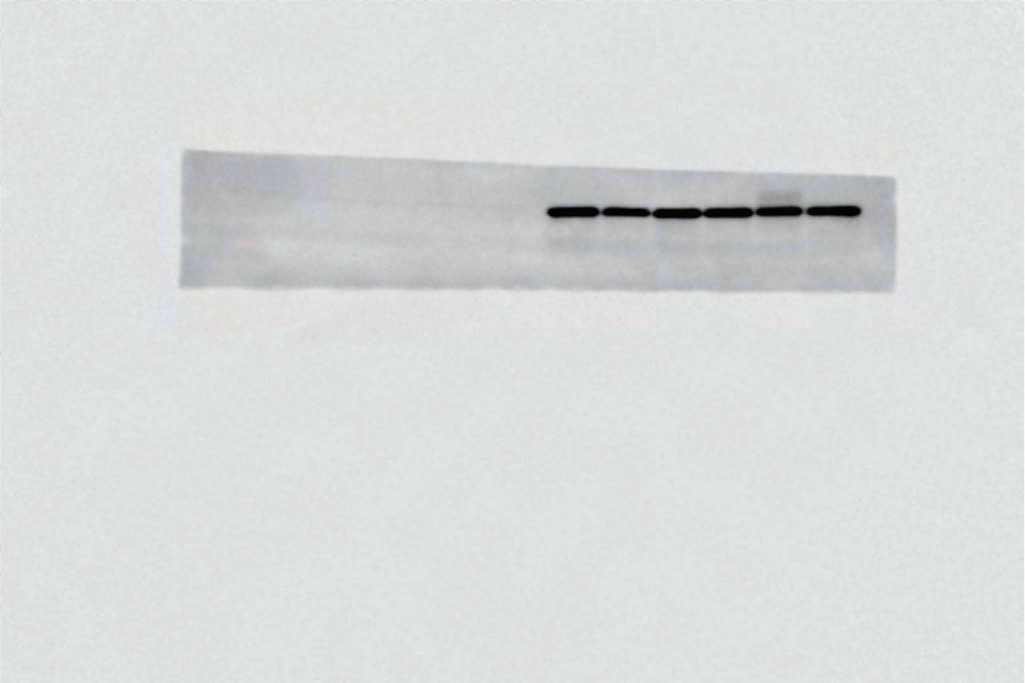

Extended Data Fig. 8g

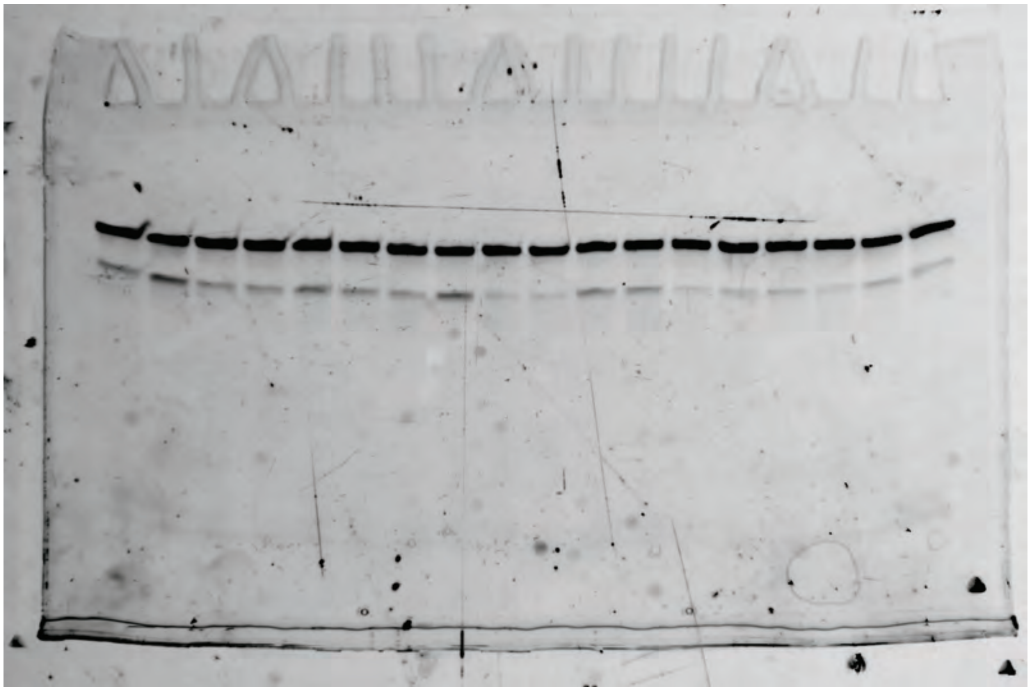

Extended Data Fig. 9b

pERK

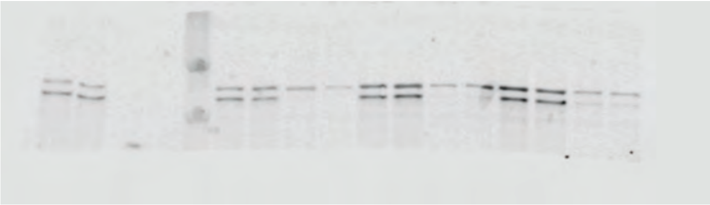

ERK

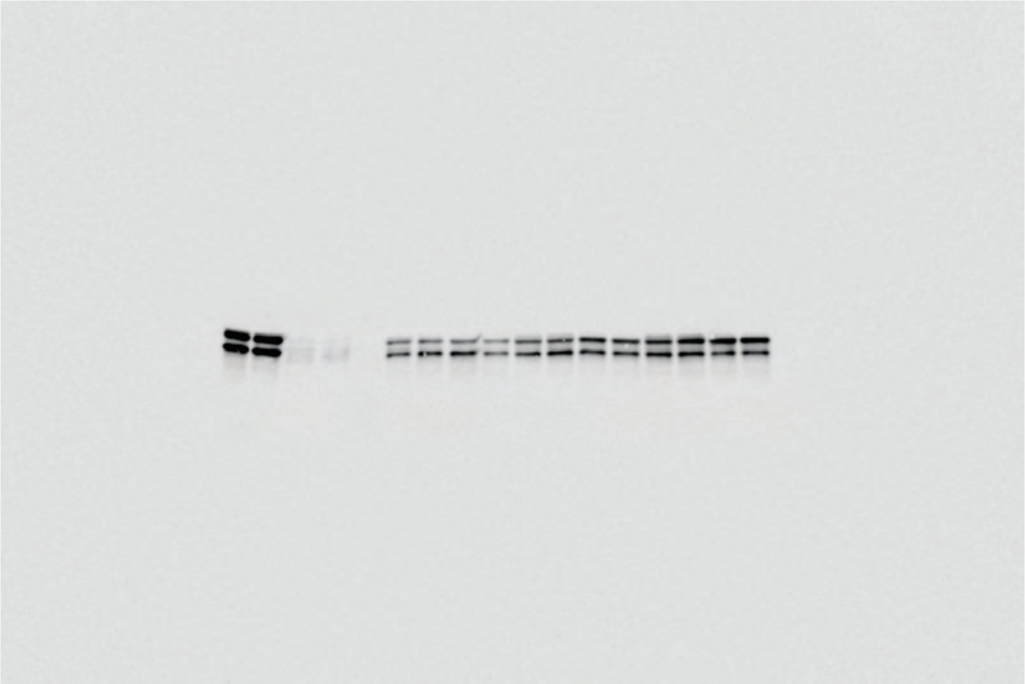

Extended Data Fig. 9b

pEGFR

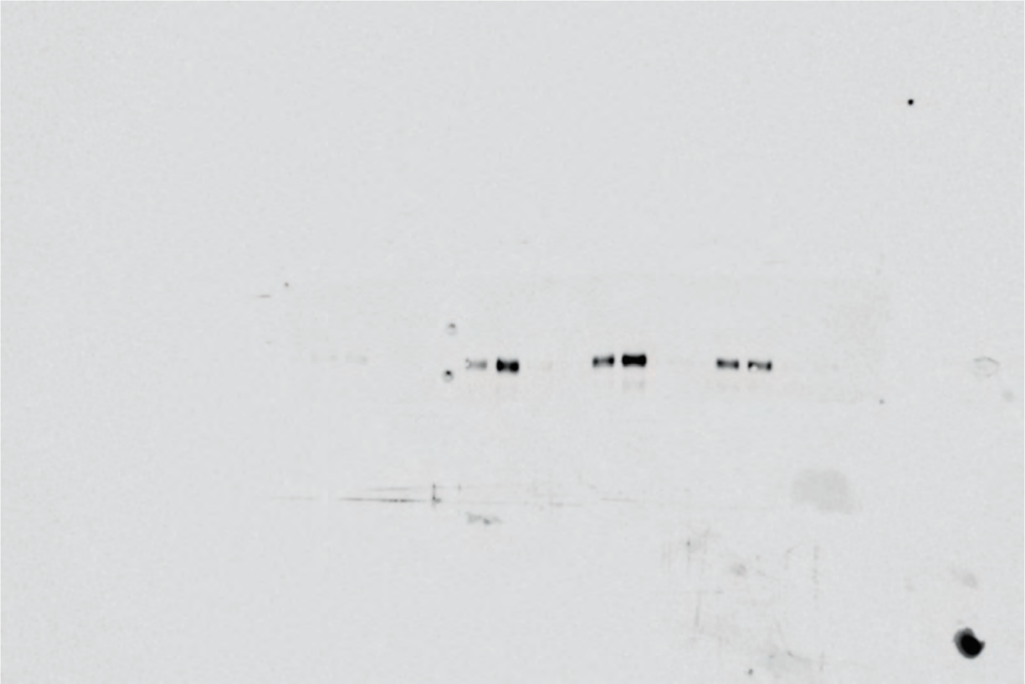

pAKT

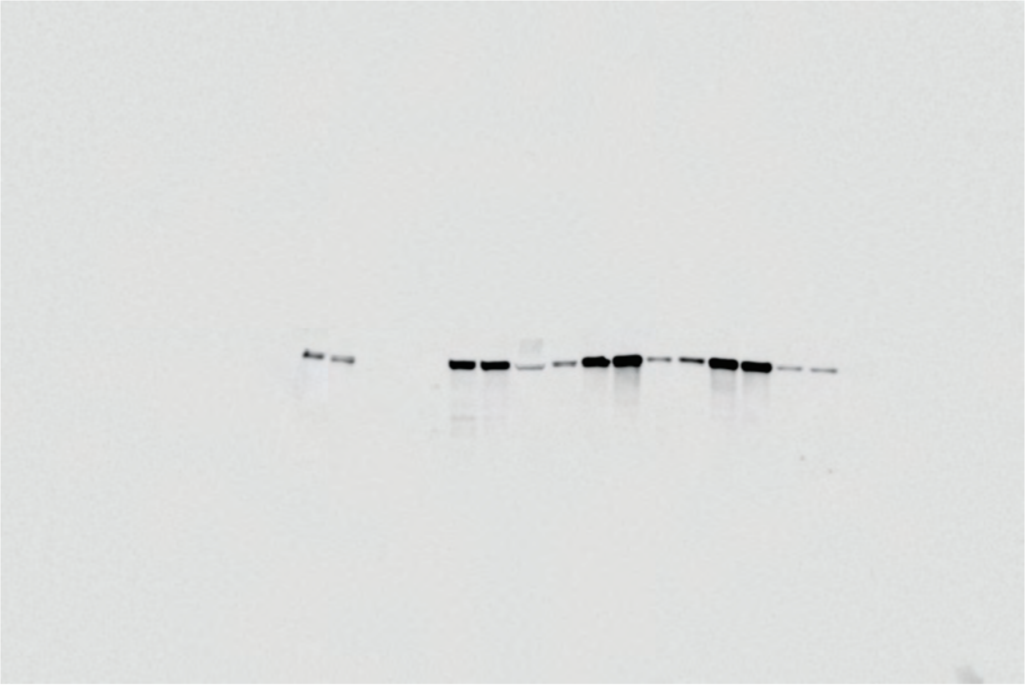

Extended Data Fig. 9b

AKT

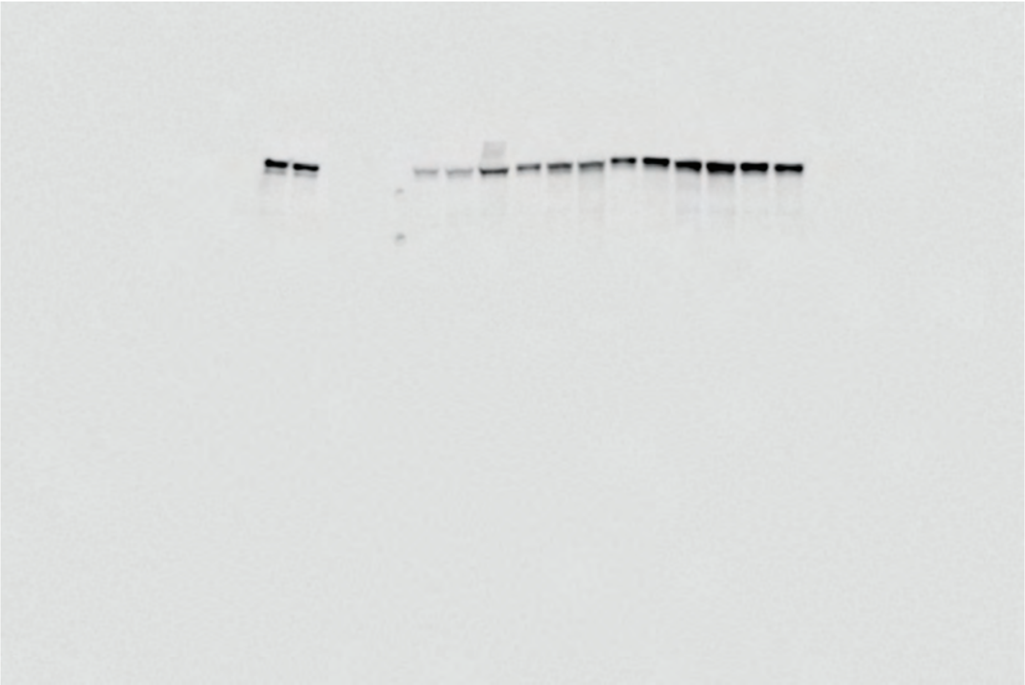

GAPDH

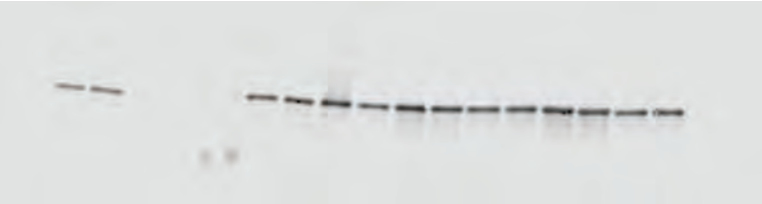

Supplement: Supplementary file 5 — Unprocessed western blots and/or gels. [file 41588_2023_1592_MOESM5_ESM.pdf]
